# Supplementary material for: Transcriptome Analysis of Intermittent Light Induced Early Bolting in Flowering Chinese Cabbage
Source: Plants (Basel). 2024 Mar 17;13(6):866. doi: 10.3390/plants13060866 (PMC10975546; doi:10.3390/plants13060866)
Supplement: Supplementary file 1 [file plants-13-00866-s001.zip › Figure S1.pdf]

Figure S1. A volcanic map of the difference genes between samples

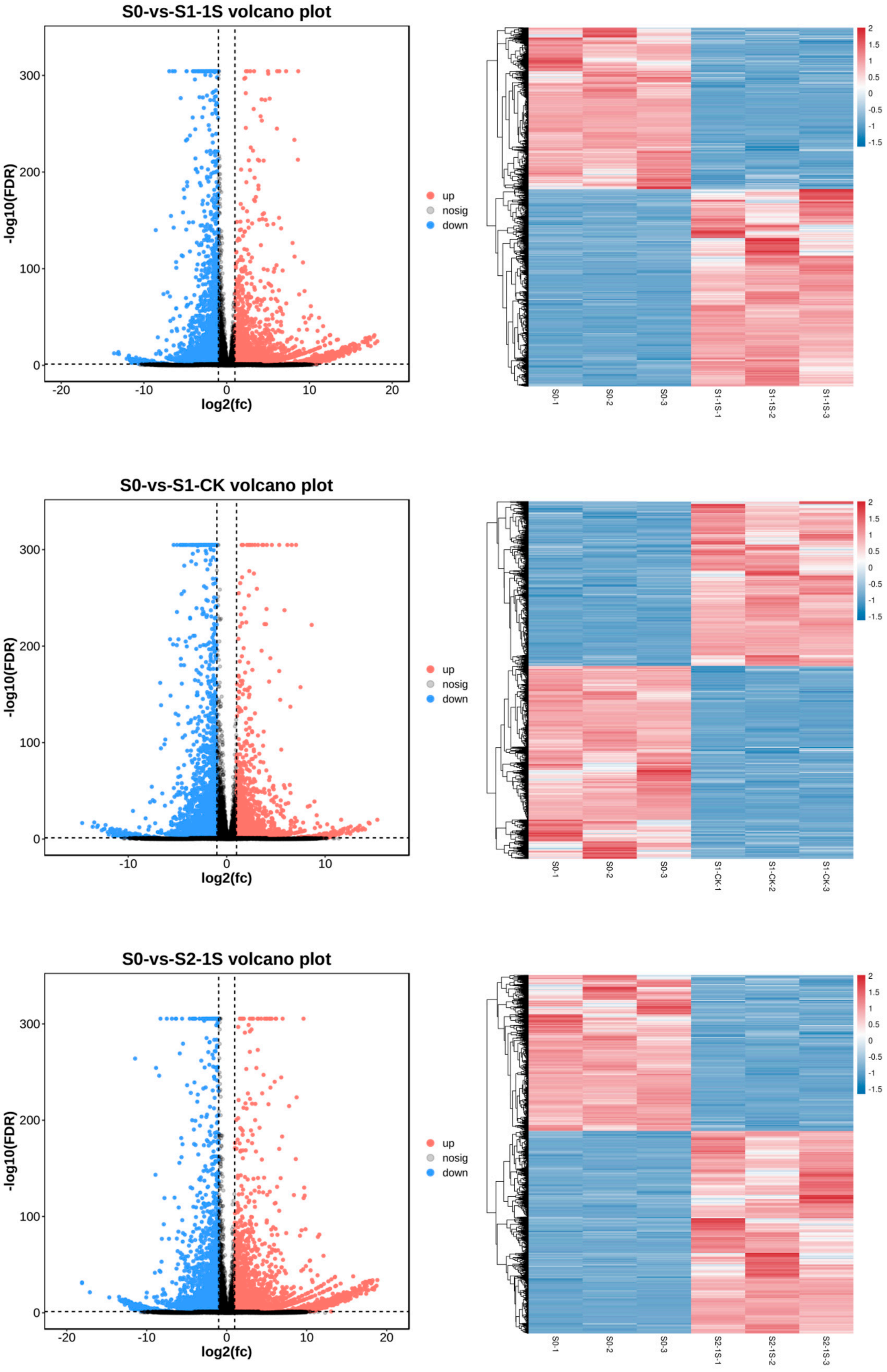

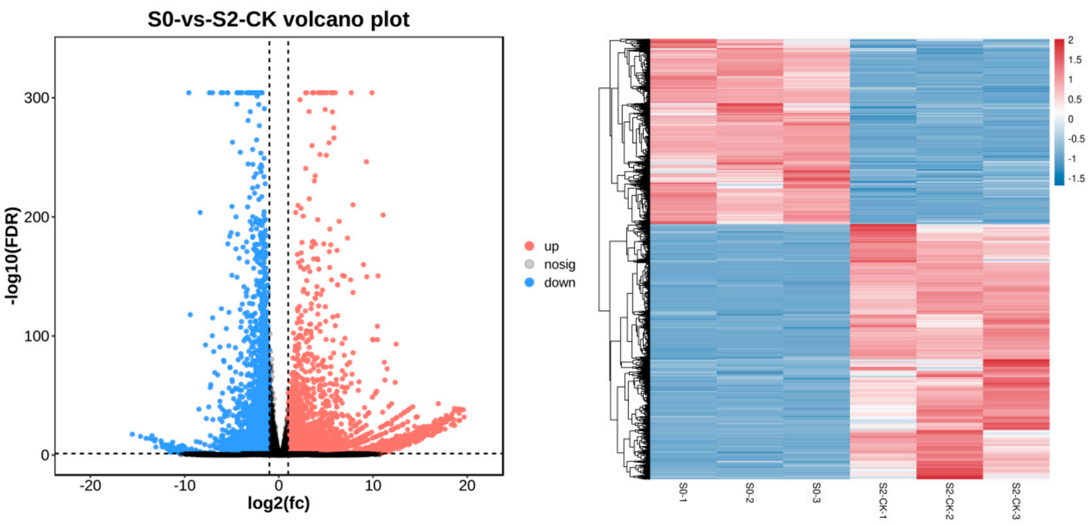
S0-vs-S2-CK volcano plot
-log10(FDR)
300
200
100
0
-20
-10
0
10
20
log2(fc)
up
nosig
down
2
1.5
1
0.5
0
-0.5
-1
-1.5
S0-1
S0-2
S0-3
S2-CK-1
S2-CK-2
S2-CK-3

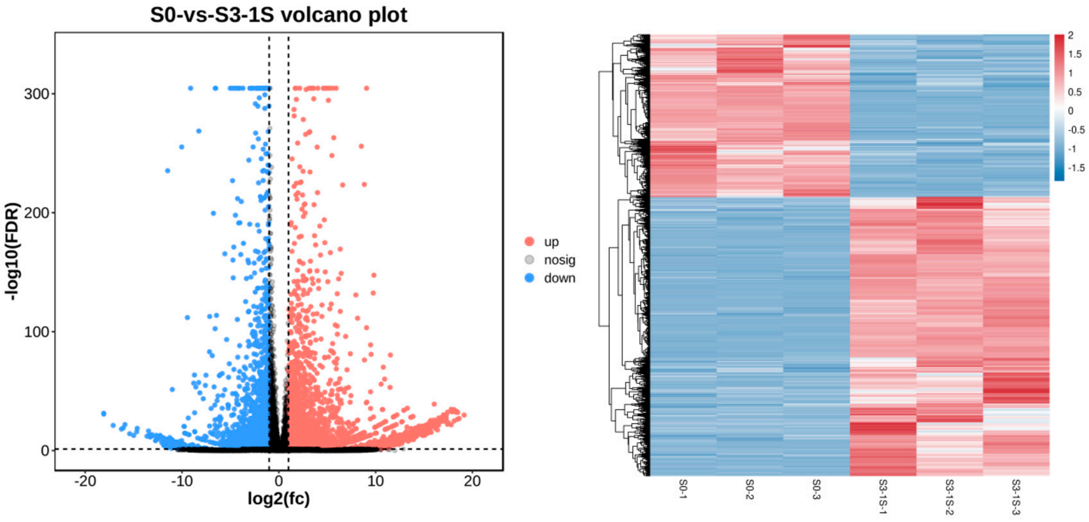
S0-vs-S3-1S volcano plot
-log10(FDR)
300
200
100
0
-20
-10
0
10
20
log2(fc)
up
nosig
down
2
1.5
1
0.5
0
-0.5
-1
-1.5
S0-1
S0-2
S0-3
S3-1S-1
S3-1S-2
S3-1S-3

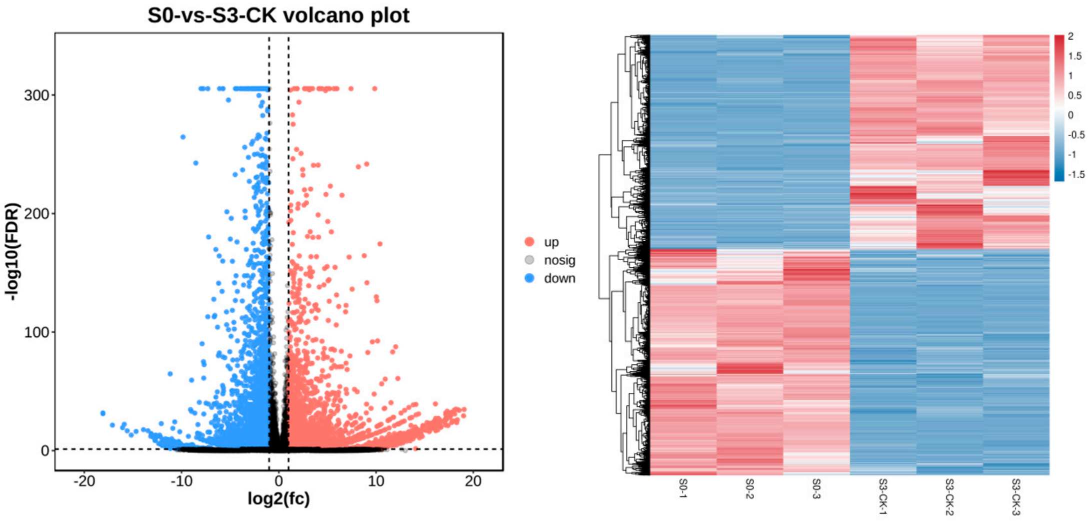
S0-vs-S3-CK volcano plot
-log10(FDR)
300
200
100
0
-20
-10
0
10
20
log2(fc)
up
nosig
down
2
1.5
1
0.5
0
-0.5
-1
-1.5
S0-1
S0-2
S0-3
S3-CK-1
S3-CK-2
S3-CK-3

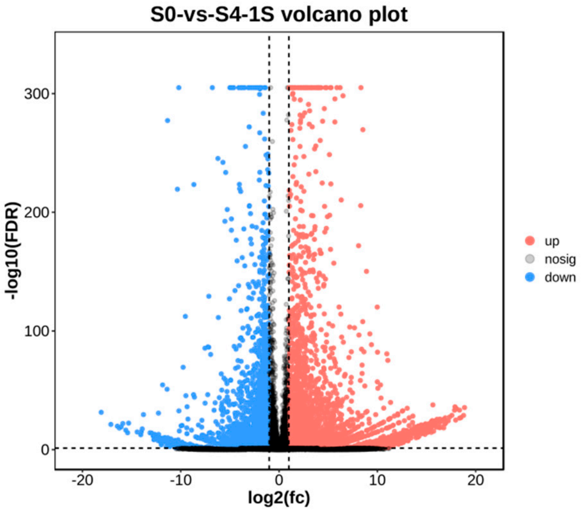
S0-vs-S4-1S volcano plot
-log10(FDR)
300
200
100
0
-20
-10
0
10
20
log2(fc)
up
nosig
down

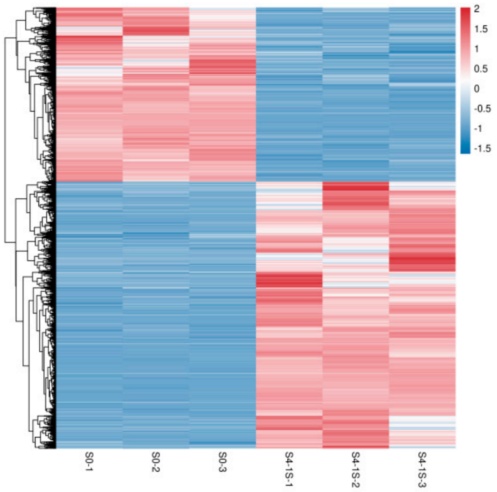
2
1.5
1
0.5
0
-0.5
-1
-1.5
S0-1
S0-2
S0-3
S4-1S-1
S4-1S-2
S4-1S-3

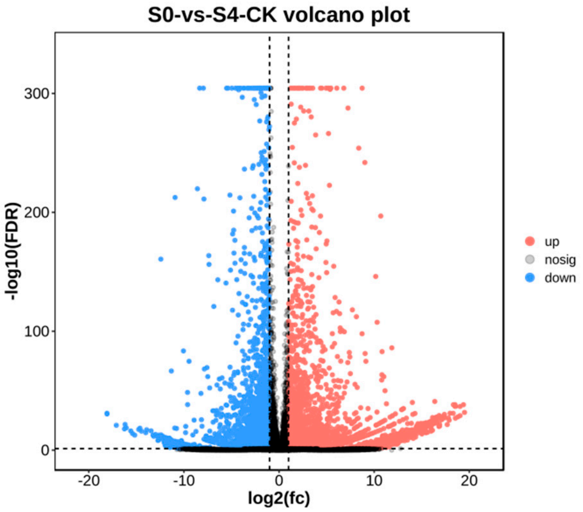
S0-vs-S4-CK volcano plot
-log10(FDR)
300
200
100
0
-20
-10
0
10
20
log2(fc)
up
nosig
down

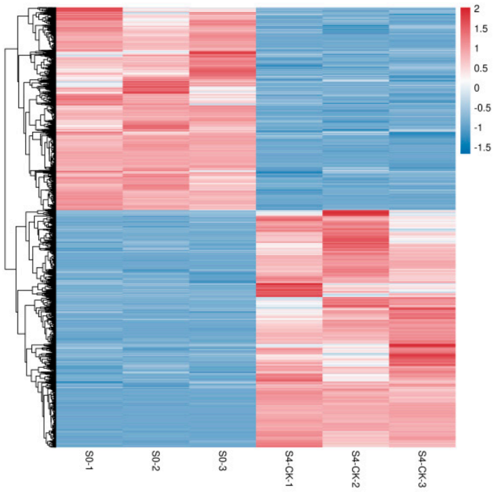
2
1.5
1
0.5
0
-0.5
-1
-1.5
S0-1
S0-2
S0-3
S4-CK-1
S4-CK-2
S4-CK-3

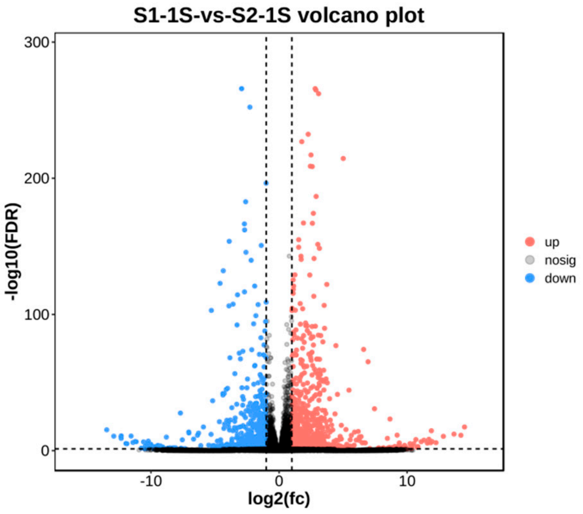
S1-1S-vs-S2-1S volcano plot
-log10(FDR)
300
200
100
0
-10
0
10
log2(fc)
up
nosig
down

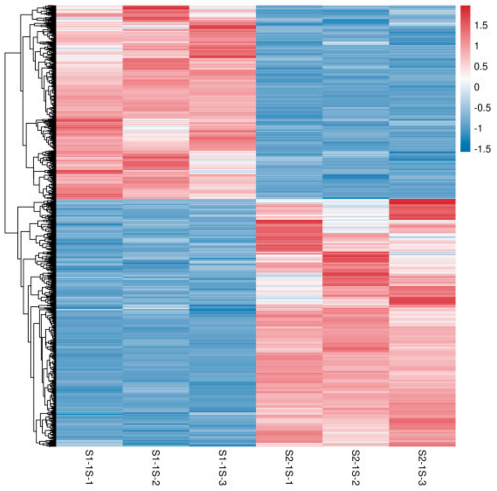
1.5
1
0.5
0
-0.5
-1
-1.5
S1-1S-1
S1-1S-2
S1-1S-3
S2-1S-1
S2-1S-2
S2-1S-3

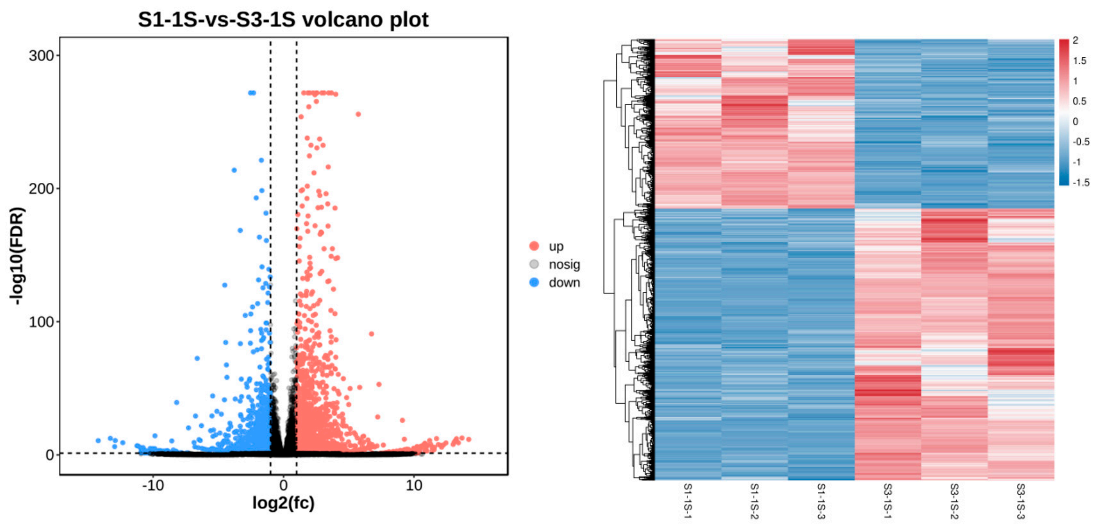
S1-1S-vs-S3-1S volcano plot
-log10(FDR)
300
200
100
0
-10
0
10
log2(fc)
up
nosig
down
2
1.5
1
0.5
0
-0.5
-1
-1.5
S1-1S-1
S1-1S-2
S1-1S-3
S3-1S-1
S3-1S-2
S3-1S-3

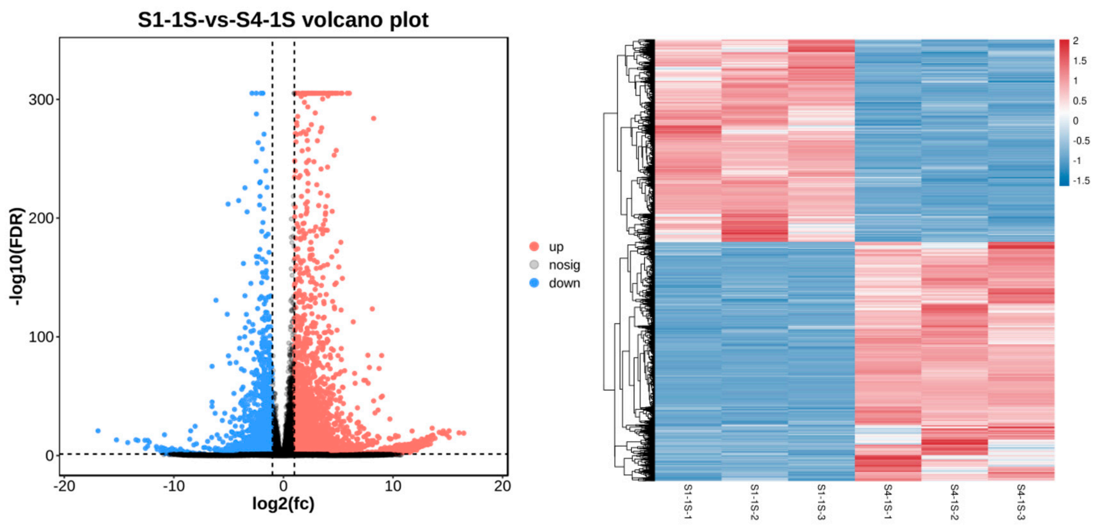
S1-1S-vs-S4-1S volcano plot
-log10(FDR)
300
200
100
0
-20
-10
0
10
20
log2(fc)
up
nosig
down
2
1.5
1
0.5
0
-0.5
-1
-1.5
S1-1S-1
S1-1S-2
S1-1S-3
S4-1S-1
S4-1S-2
S4-1S-3

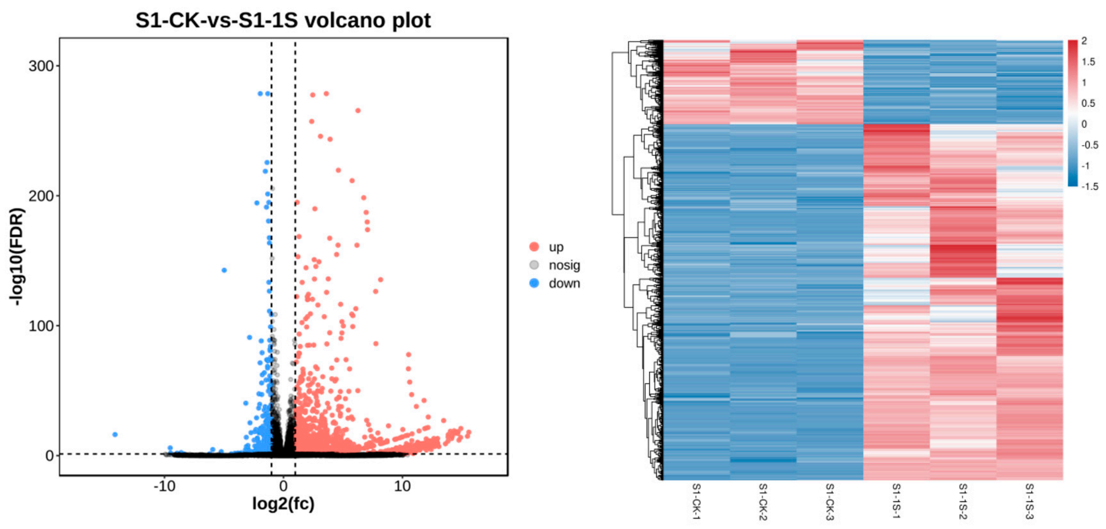
S1-CK-vs-S1-1S volcano plot
-log10(FDR)
300
200
100
0
-10
0
10
log2(fc)
up
nosig
down
2
1.5
1
0.5
0
-0.5
-1
-1.5
S1-CK-1
S1-CK-2
S1-CK-3
S1-1S-1
S1-1S-2
S1-1S-3

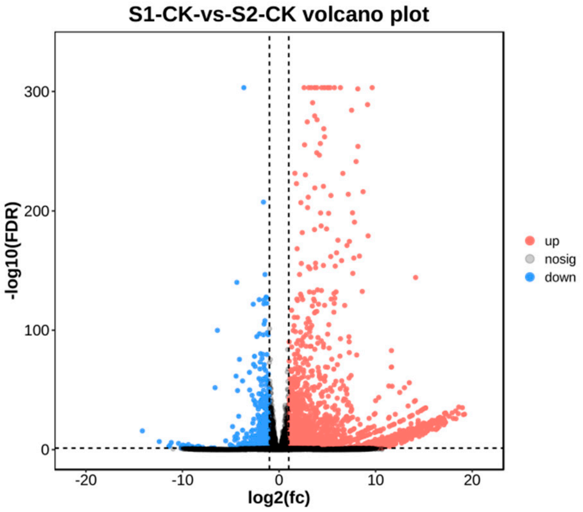
S1-CK-vs-S2-CK volcano plot
-log10(FDR)
log2(fc)
up
nosig
down

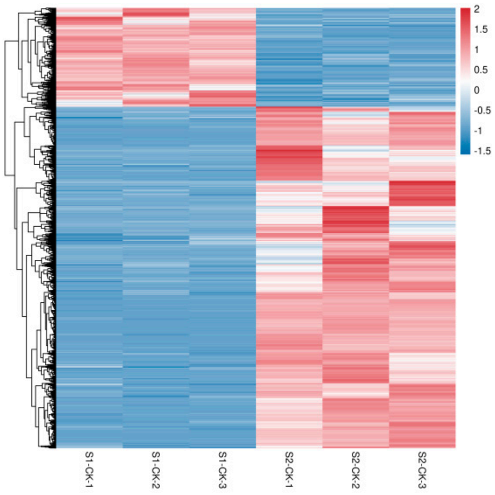
S1-CK-1
S1-CK-2
S1-CK-3
S2-CK-1
S2-CK-2
S2-CK-3

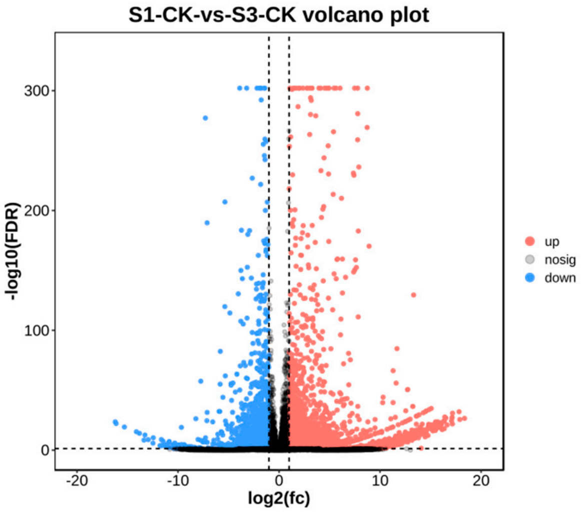
S1-CK-vs-S3-CK volcano plot
-log10(FDR)
log2(fc)
up
nosig
down

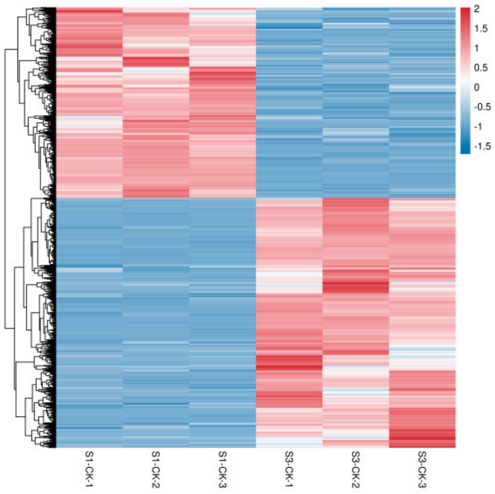
S1-CK-1
S1-CK-2
S1-CK-3
S3-CK-1
S3-CK-2
S3-CK-3

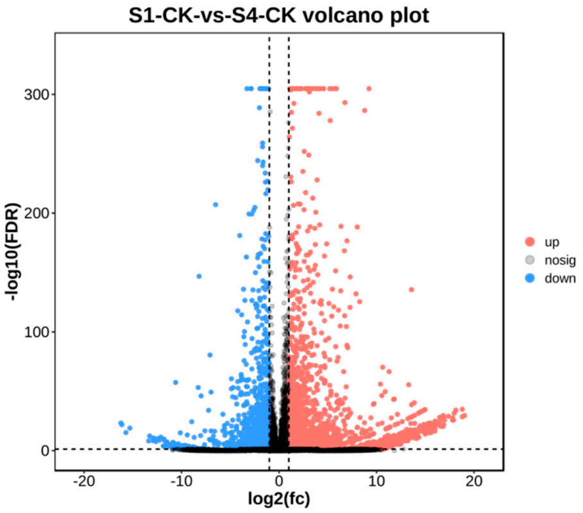
S1-CK-vs-S4-CK volcano plot
-log10(FDR)
log2(fc)
up
nosig
down

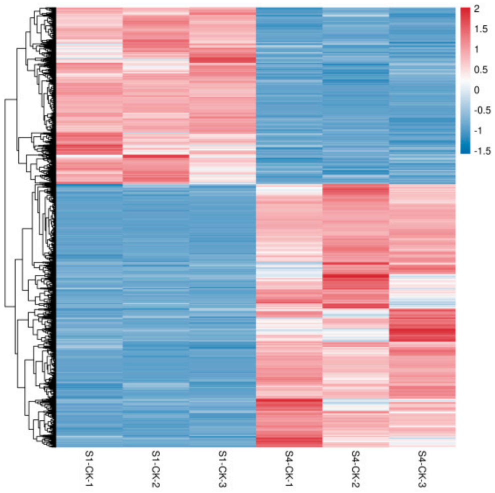
S1-CK-1
S1-CK-2
S1-CK-3
S4-CK-1
S4-CK-2
S4-CK-3

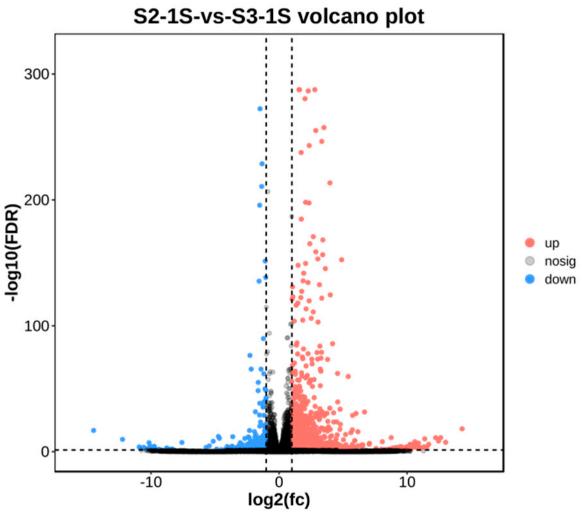
S2-1S-vs-S3-1S volcano plot
-log10(FDR)
log2(fc)
up
nosig
down

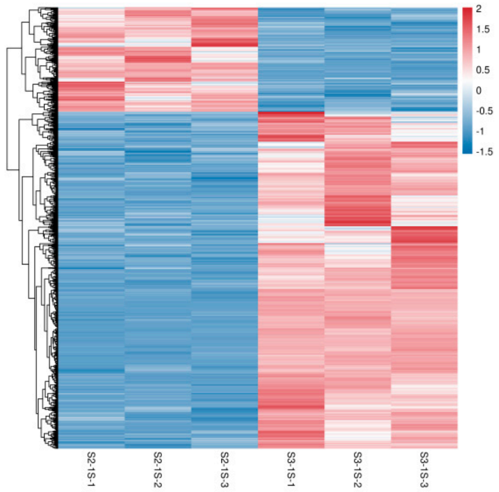
S2-1S-1
S2-1S-2
S2-1S-3
S3-1S-1
S3-1S-2
S3-1S-3

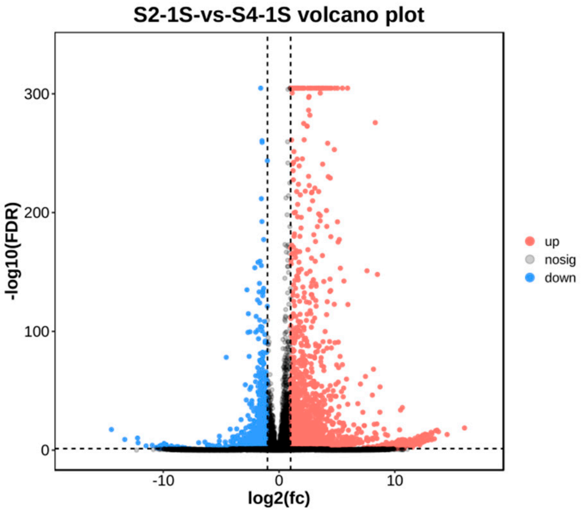
S2-1S-vs-S4-1S volcano plot
-log10(FDR)
log2(fc)
up
nosig
down

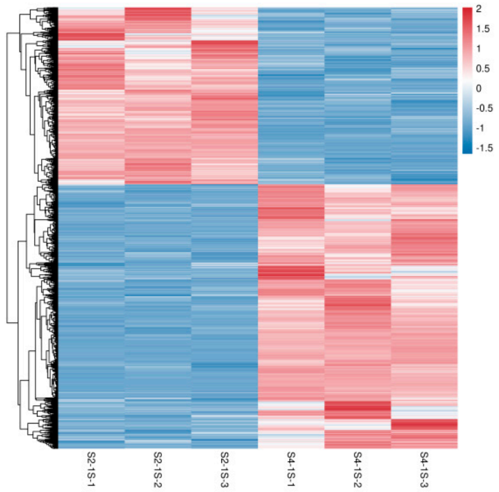
S2-1S-1
S2-1S-2
S2-1S-3
S4-1S-1
S4-1S-2
S4-1S-3

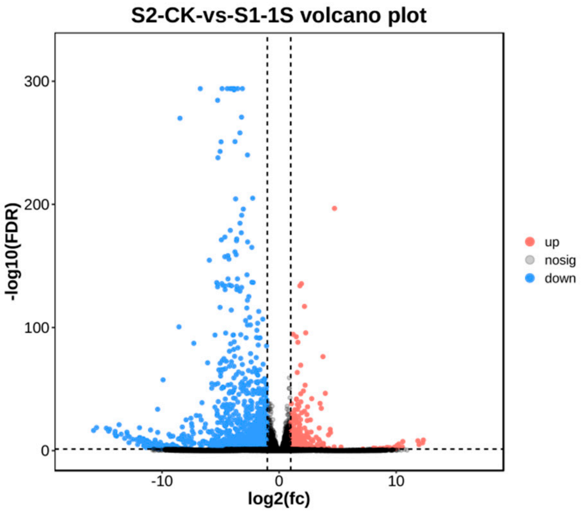
S2-CK-vs-S1-1S volcano plot
-log10(FDR)
log2(fc)
up
nosig
down

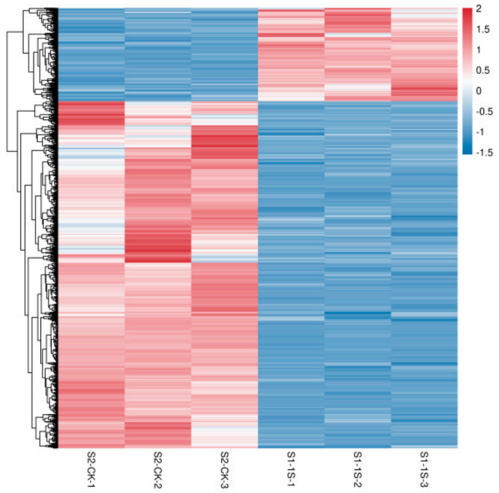
S2-CK-1
S2-CK-2
S2-CK-3
S1-1S-1
S1-1S-2
S1-1S-3

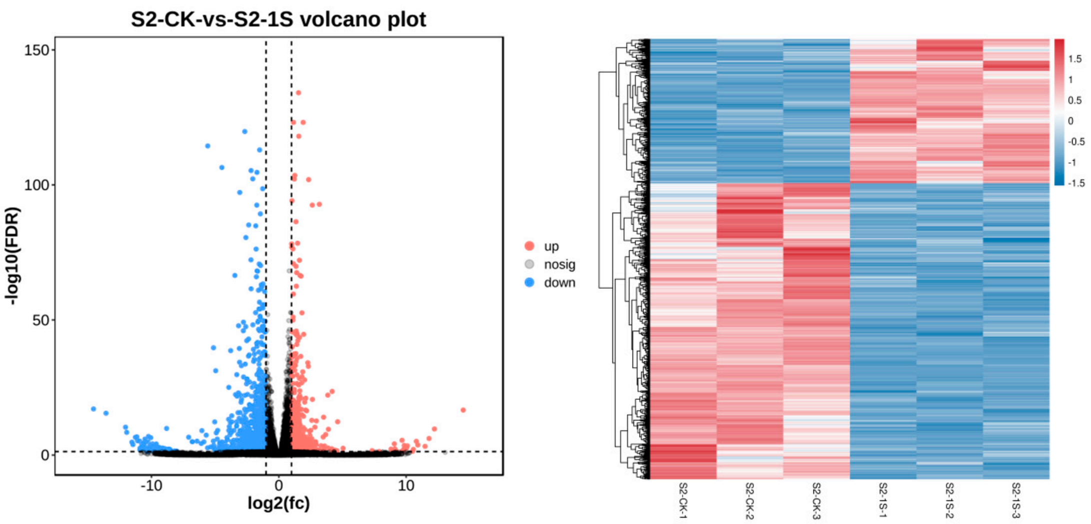
S2-CK-vs-S2-1S volcano plot
-log10(FDR)
150
100
50
0
-10
0
10
log2(fc)
up
nosig
down
1.5
1
0.5
0
-0.5
-1
-1.5
S2-CK-1
S2-CK-2
S2-CK-3
S2-1S-1
S2-1S-2
S2-1S-3

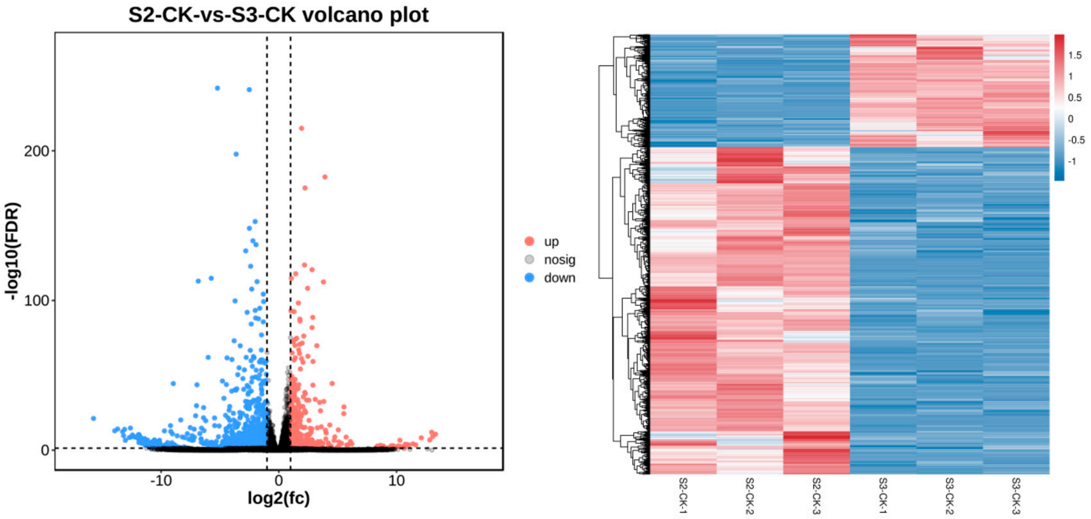
S2-CK-vs-S3-CK volcano plot
-log10(FDR)
200
100
0
-10
0
10
log2(fc)
up
nosig
down
1.5
1
0.5
0
-0.5
-1
S2-CK-1
S2-CK-2
S2-CK-3
S3-CK-1
S3-CK-2
S3-CK-3

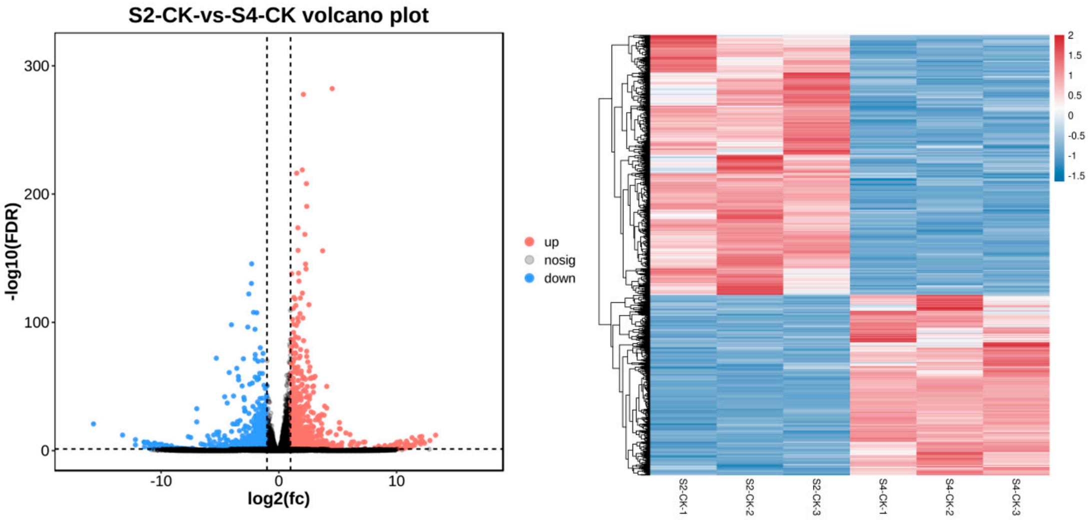
S2-CK-vs-S4-CK volcano plot
-log10(FDR)
300
200
100
0
-10
0
10
log2(fc)
up
nosig
down
2
1.5
1
0.5
0
-0.5
-1
-1.5
S2-CK-1
S2-CK-2
S2-CK-3
S4-CK-1
S4-CK-2
S4-CK-3

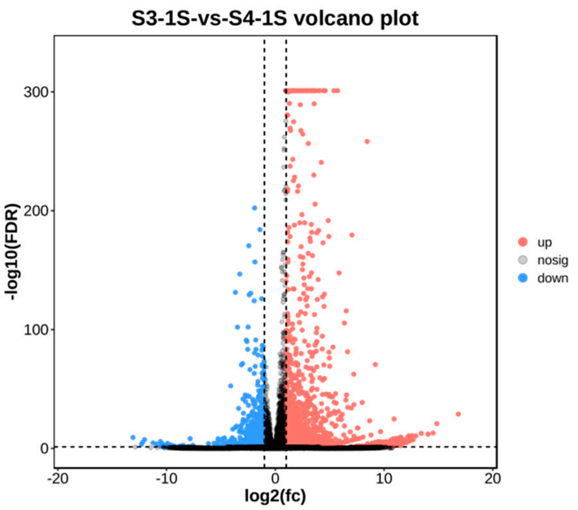
S3-1S-vs-S4-1S volcano plot
-log10(FDR)
log2(fc)
up
nosig
down

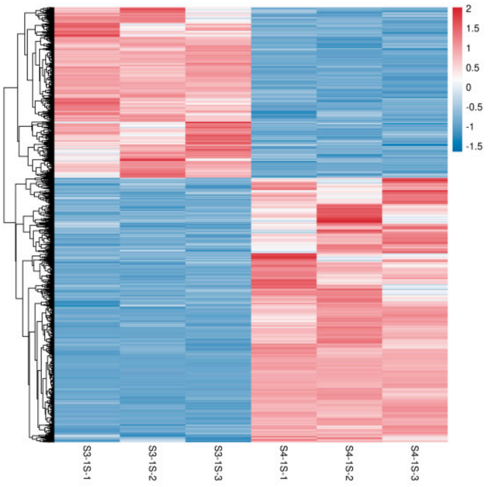
S3-1S-1
S3-1S-2
S3-1S-3
S4-1S-1
S4-1S-2
S4-1S-3

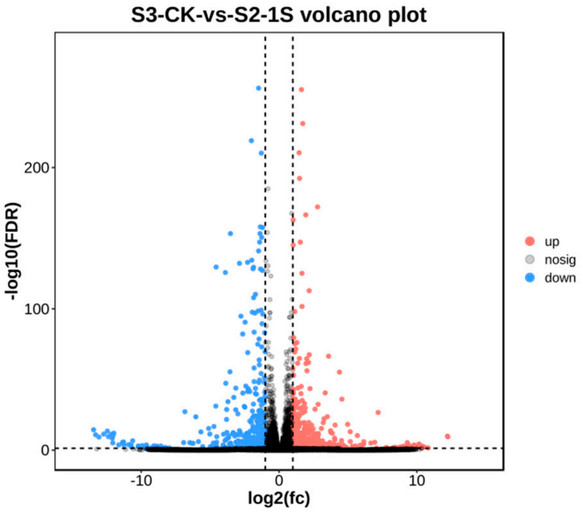
S3-CK-vs-S2-1S volcano plot
-log10(FDR)
log2(fc)
up
nosig
down

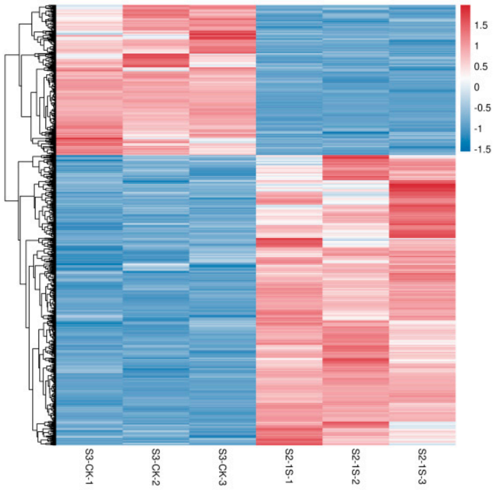
S3-CK-1
S3-CK-2
S3-CK-3
S2-1S-1
S2-1S-2
S2-1S-3

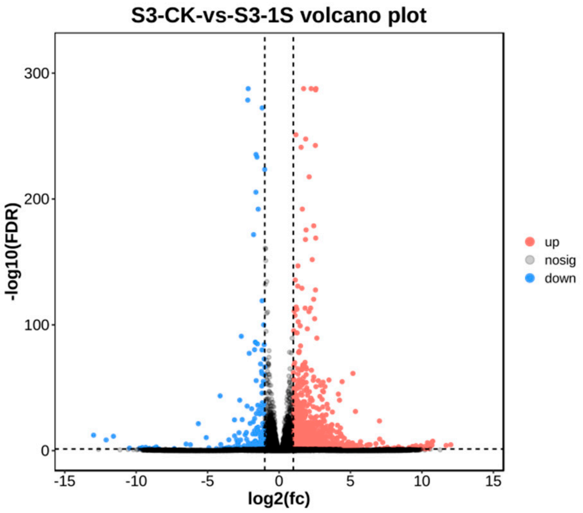
S3-CK-vs-S3-1S volcano plot
-log10(FDR)
log2(fc)
up
nosig
down

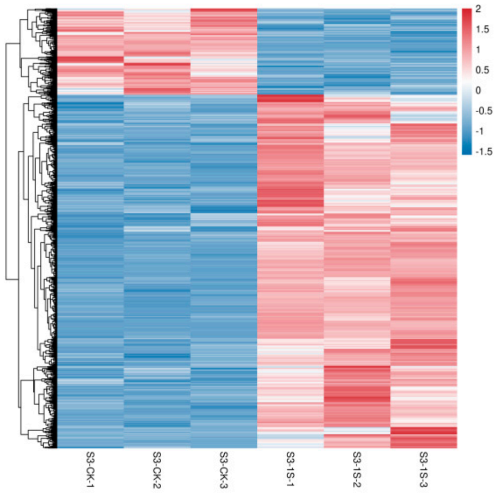
S3-CK-1
S3-CK-2
S3-CK-3
S3-1S-1
S3-1S-2
S3-1S-3

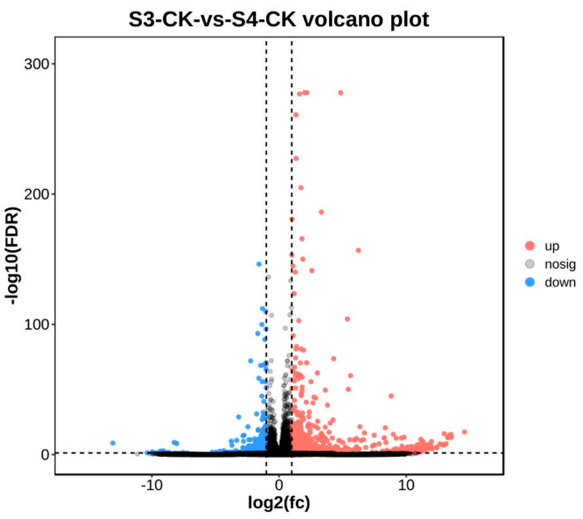
S3-CK-vs-S4-CK volcano plot
-log10(FDR)
log2(fc)
300
200
100
0
-10
0
10
up
nosig
down

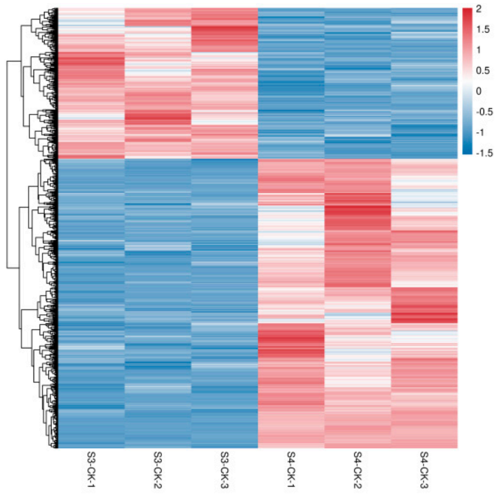
S3-CK-1
S3-CK-2
S3-CK-3
S4-CK-1
S4-CK-2
S4-CK-3
2
1.5
1
0.5
0
-0.5
-1
-1.5

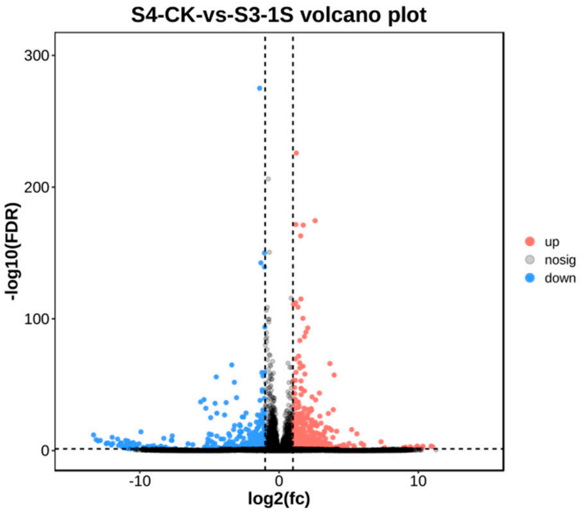
S4-CK-vs-S3-1S volcano plot
-log10(FDR)
log2(fc)
300
200
100
0
-10
0
10
up
nosig
down

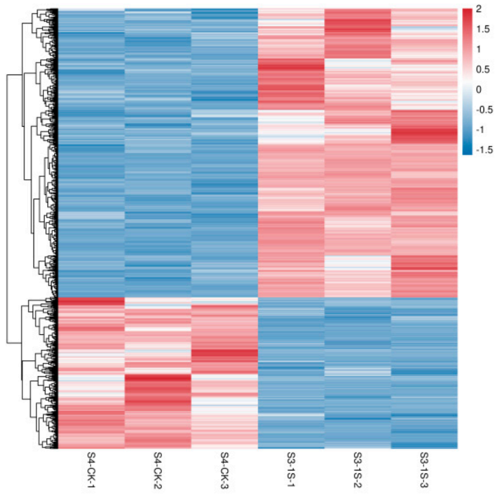
S4-CK-1
S4-CK-2
S4-CK-3
S3-1S-1
S3-1S-2
S3-1S-3
2
1.5
1
0.5
0
-0.5
-1
-1.5

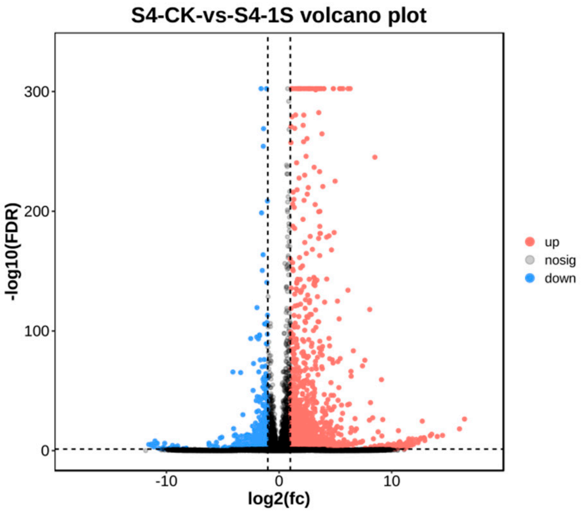
S4-CK-vs-S4-1S volcano plot
-log10(FDR)
log2(fc)
300
200
100
0
-10
0
10
up
nosig
down

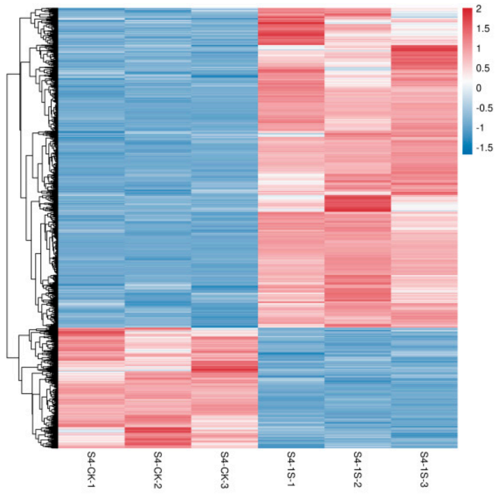
S4-CK-1
S4-CK-2
S4-CK-3
S4-1S-1
S4-1S-2
S4-1S-3
2
1.5
1
0.5
0
-0.5
-1
-1.5
