# Supplementary material for: Transcriptome Analysis of Intermittent Light Induced Early Bolting in Flowering Chinese Cabbage
Source: Plants (Basel). 2024 Mar 17;13(6):866. doi: 10.3390/plants13060866 (PMC10975546; doi:10.3390/plants13060866)
Supplement: Supplementary file 1 [file plants-13-00866-s001.zip › Figure S2.pdf]

Figure S2. GO Rich Map of DEGs Between Sample

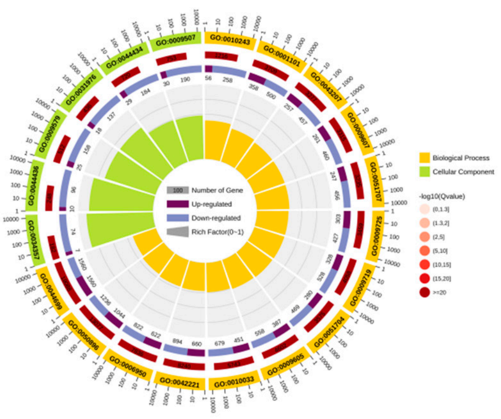

S0 vs S1-1s

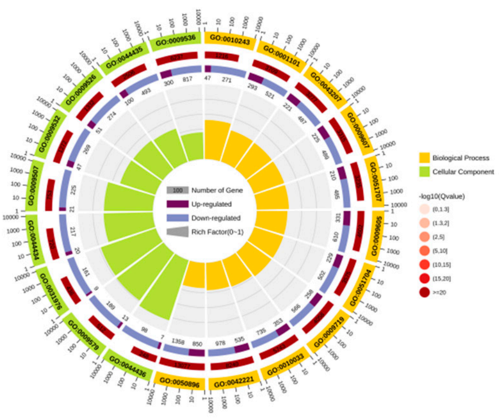

S0 vs S1-CK

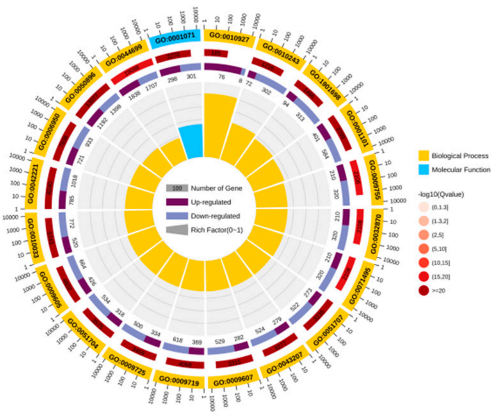

S0 vs S2-1s

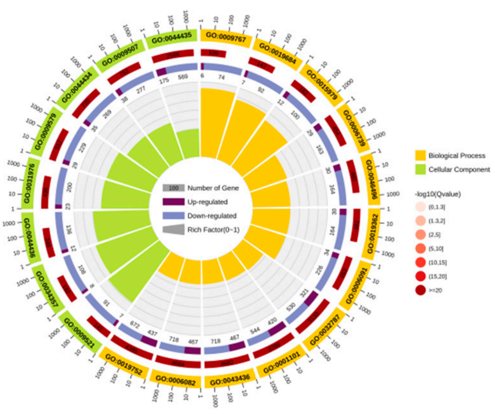

S0 vs S2-CK

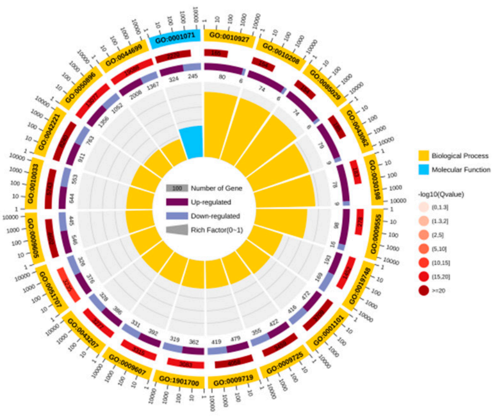

S0 vs S3-1s

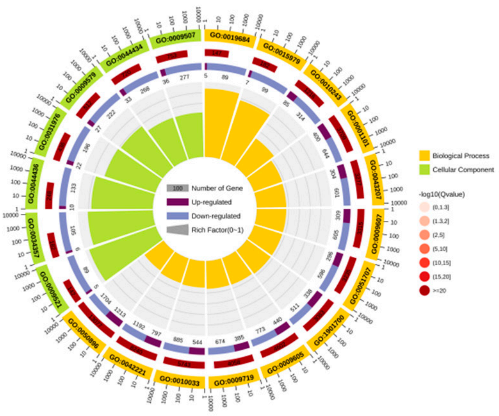

S0 vs S3-CK

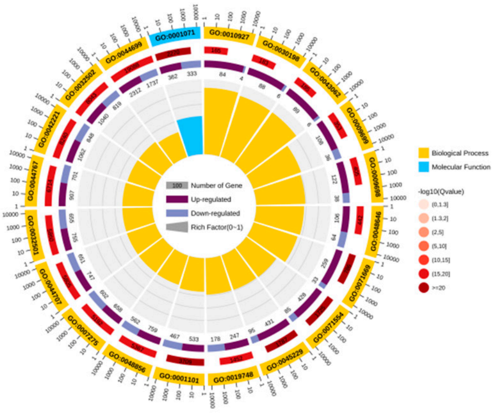

S0 vs S4-1s

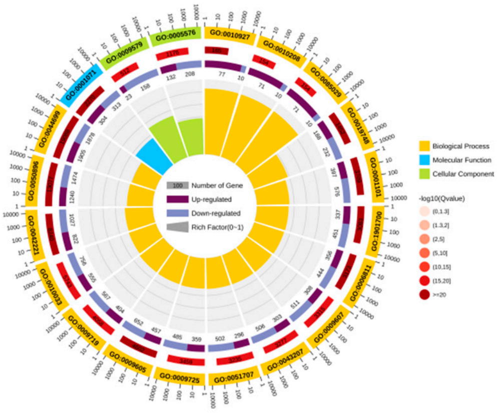

S0 vs S4-CK

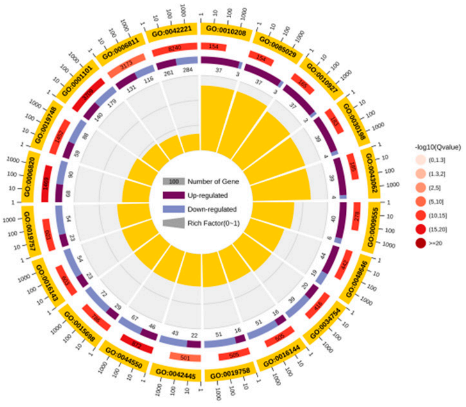

S1-1s vs S2-1s

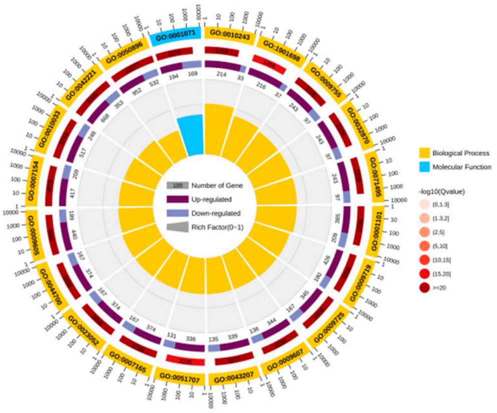

S1-1s vs S3-1s

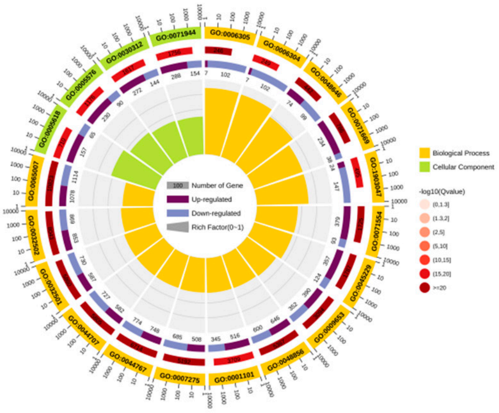

S1-1s vs S4-1s

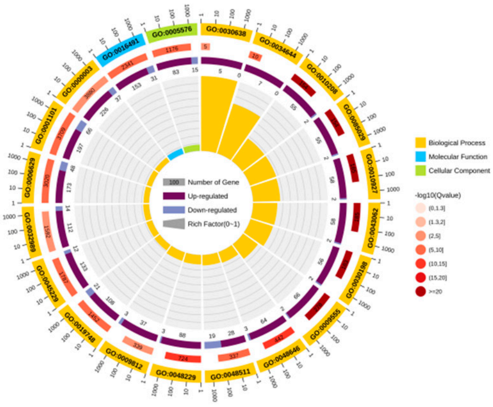

S1-CK vs S1-1s

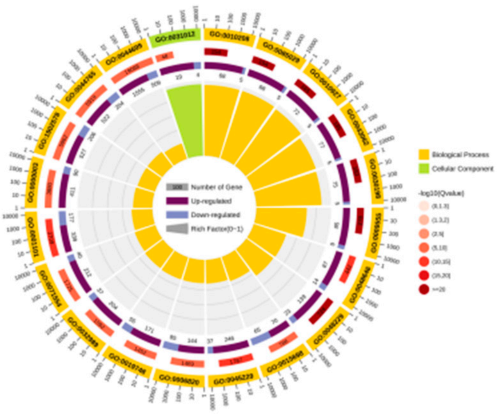

S1-CK vs S2-CK

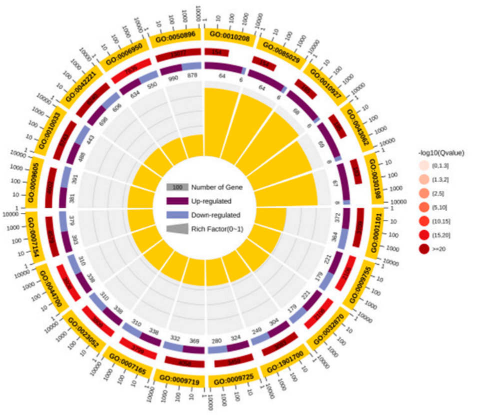

S1-CK vs S3-CK

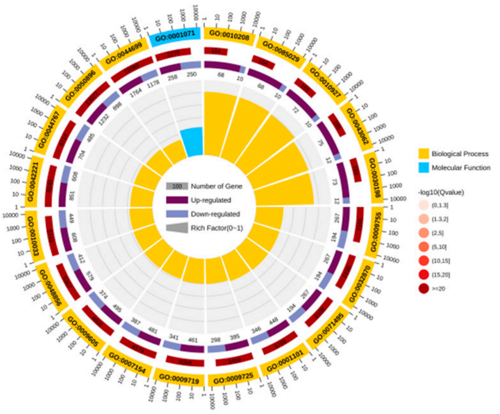

S1-CK vs S4-CK

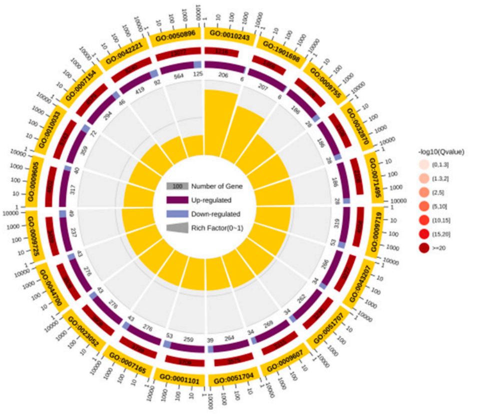

S2-1s vs S3-1s

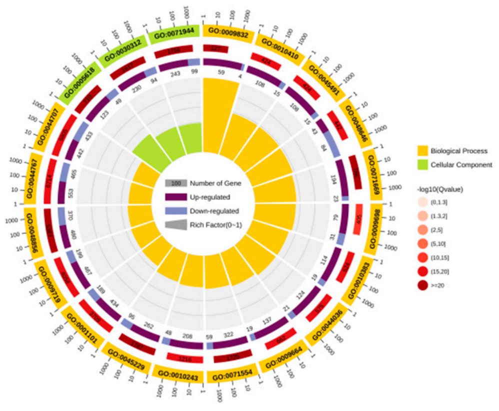

S2-1s vs S4-1s

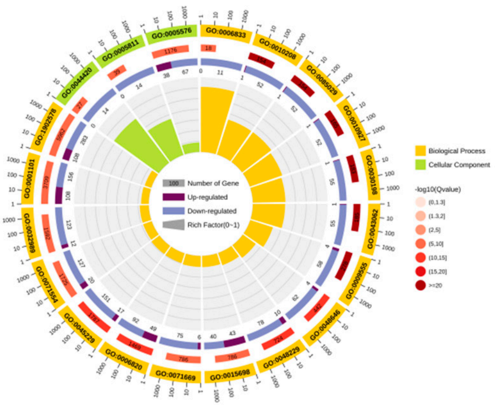

S2-CK vs S1-1s

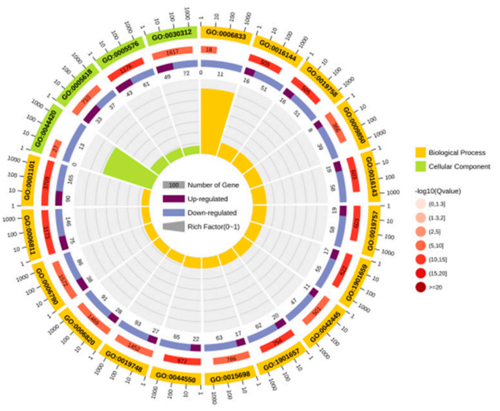

S2-CK vs S2-1s

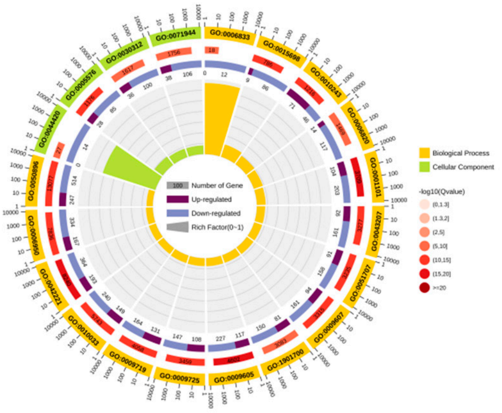

S2-CK vs S3-CK

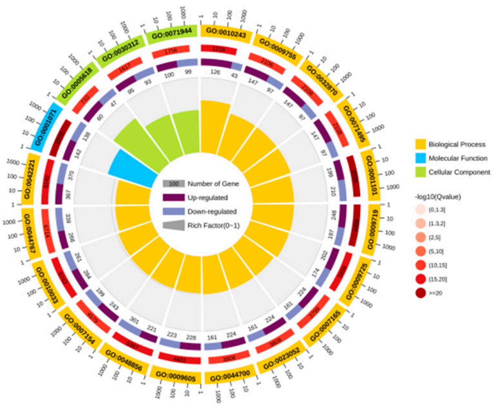

S2-CK vs S4-CK

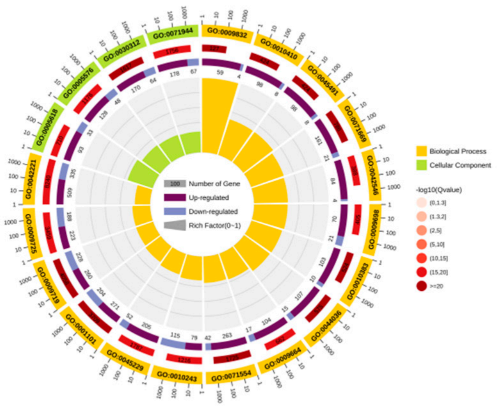

S3-1s vs S4-1s

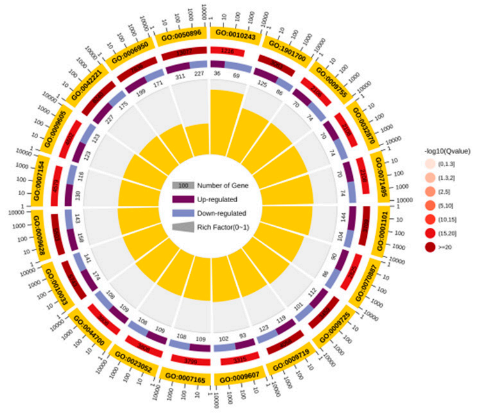

S3-CK vs S2-1s

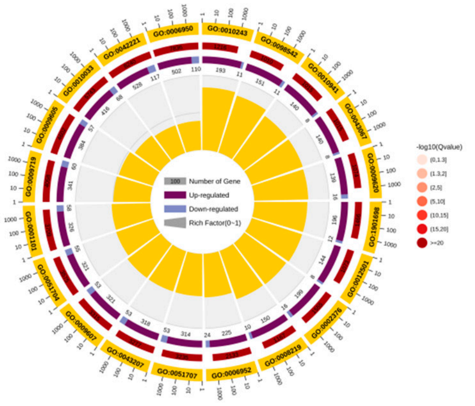

S3-CK vs S3-1s

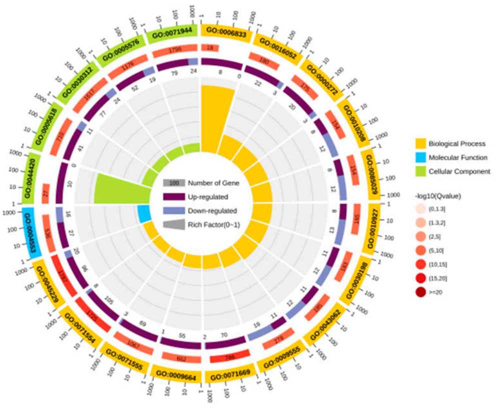

S3-CK vs S4-CK

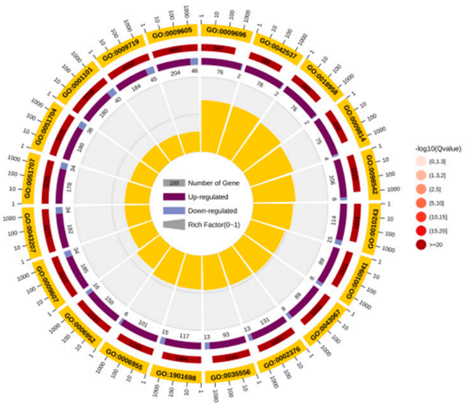

S4-CK vs S3-1s

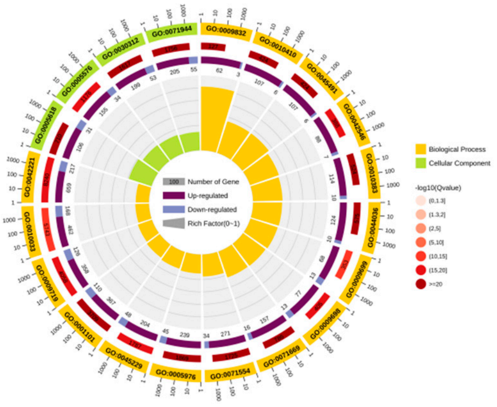

S4-CK vs S4-1s
