# Supplementary material for: Transcriptome Analysis of Intermittent Light Induced Early Bolting in Flowering Chinese Cabbage
Source: Plants (Basel). 2024 Mar 17;13(6):866. doi: 10.3390/plants13060866 (PMC10975546; doi:10.3390/plants13060866)
Supplement: Supplementary file 1 [file plants-13-00866-s001.zip › Figure S4.pdf]

Figure S4. GO Concentration Map of DEGs Between 18 Modules

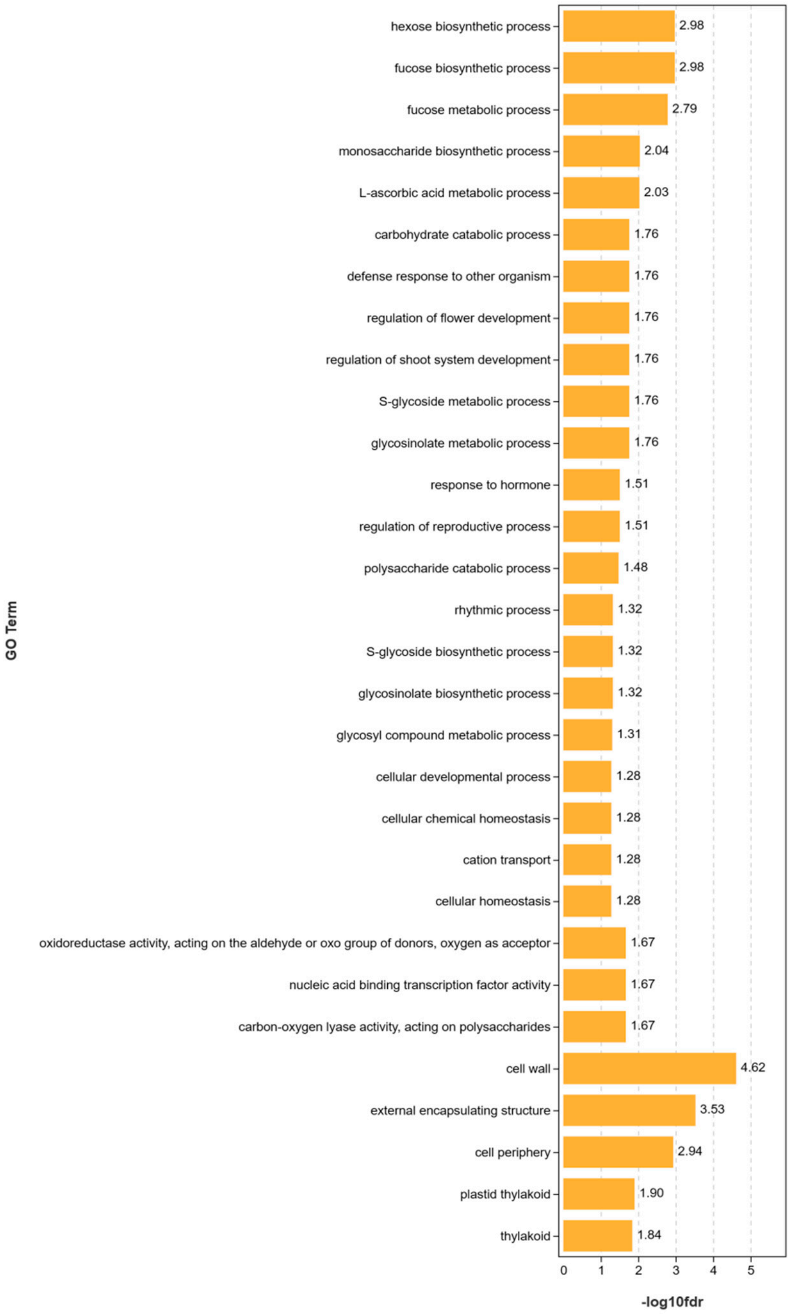

MM1.green

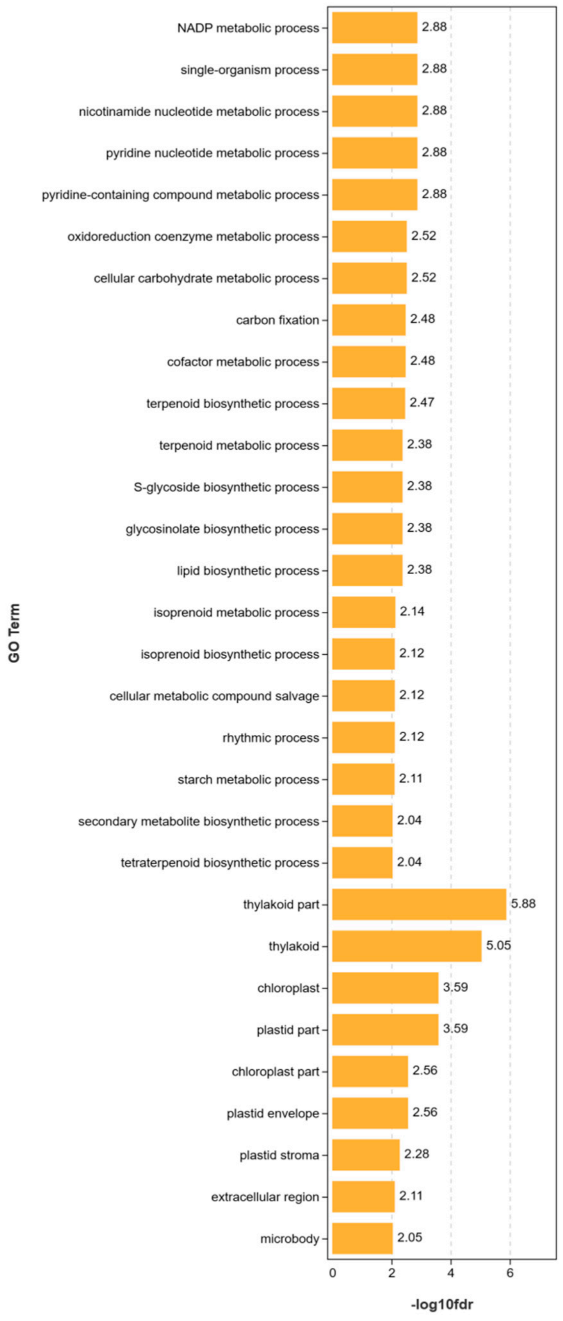
GO Term
NADP metabolic process
2.88
single-organism process
2.88
nicotinamide nucleotide metabolic process
2.88
pyridine nucleotide metabolic process
2.88
pyridine-containing compound metabolic process
2.88
oxidoreduction coenzyme metabolic process
2.52
cellular carbohydrate metabolic process
2.52
carbon fixation
2.48
cofactor metabolic process
2.48
terpenoid biosynthetic process
2.47
terpenoid metabolic process
2.38
S-glycoside biosynthetic process
2.38
glycosinolate biosynthetic process
2.38
lipid biosynthetic process
2.38
isoprenoid metabolic process
2.14
isoprenoid biosynthetic process
2.12
cellular metabolic compound salvage
2.12
rhythmic process
2.12
starch metabolic process
2.11
secondary metabolite biosynthetic process
2.04
tetraterpenoid biosynthetic process
2.04
thylakoid part
5.88
thylakoid
5.05
chloroplast
3.59
plastid part
3.59
chloroplast part
2.56
plastid envelope
2.56
plastid stroma
2.28
extracellular region
2.11
microbody
2.05
0
2
4
6
-log10fdr

MM2.purple

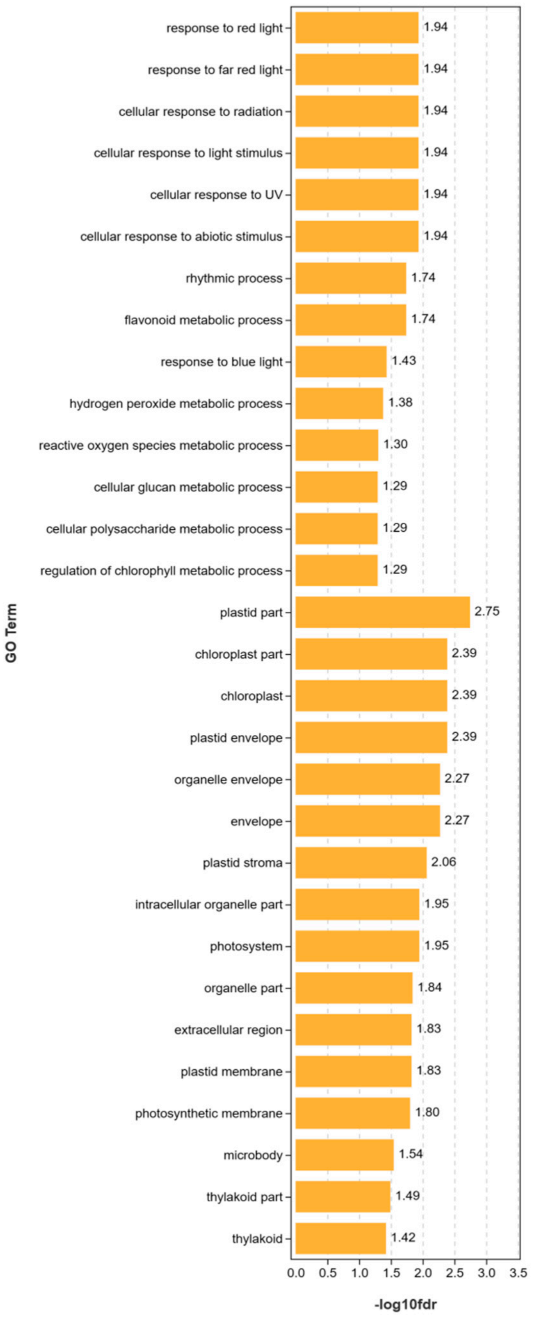
response to red light 1.94
response to far red light 1.94
cellular response to radiation 1.94
cellular response to light stimulus 1.94
cellular response to UV 1.94
cellular response to abiotic stimulus 1.94
rhythmic process 1.74
flavonoid metabolic process 1.74
response to blue light 1.43
hydrogen peroxide metabolic process 1.38
reactive oxygen species metabolic process 1.30
cellular glucan metabolic process 1.29
cellular polysaccharide metabolic process 1.29
regulation of chlorophyll metabolic process 1.29
plastid part 2.75
chloroplast part 2.39
chloroplast 2.39
plastid envelope 2.39
organelle envelope 2.27
envelope 2.27
plastid stroma 2.06
intracellular organelle part 1.95
photosystem 1.95
organelle part 1.84
extracellular region 1.83
plastid membrane 1.83
photosynthetic membrane 1.80
microbody 1.54
thylakoid part 1.49
thylakoid 1.42
GO Term
0.0 0.5 1.0 1.5 2.0 2.5 3.0 3.5
-log10fdr

MM3.darkred

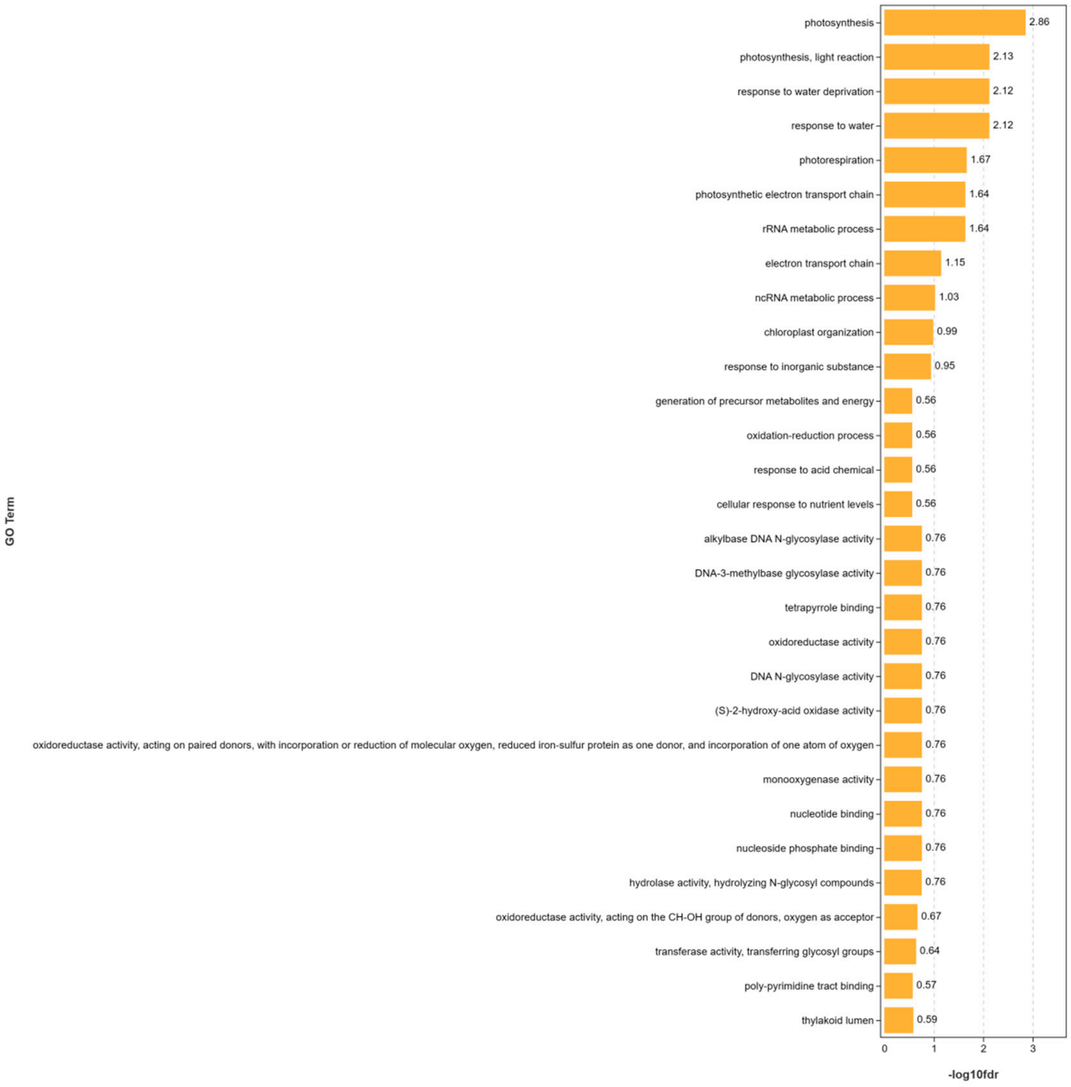
GO Term
photosynthesis 2.86
photosynthesis, light reaction 2.13
response to water deprivation 2.12
response to water 2.12
photorespiration 1.67
photosynthetic electron transport chain 1.64
rRNA metabolic process 1.64
electron transport chain 1.15
ncRNA metabolic process 1.03
chloroplast organization 0.99
response to inorganic substance 0.95
generation of precursor metabolites and energy 0.56
oxidation-reduction process 0.56
response to acid chemical 0.56
cellular response to nutrient levels 0.56
alkylbase DNA N-glycosylase activity 0.76
DNA-3-methylbase glycosylase activity 0.76
tetrapyrrole binding 0.76
oxidoreductase activity 0.76
DNA N-glycosylase activity 0.76
(S)-2-hydroxy-acid oxidase activity 0.76
oxidoreductase activity, acting on paired donors, with incorporation or reduction of molecular oxygen, reduced iron-sulfur protein as one donor, and incorporation of one atom of oxygen 0.76
monooxygenase activity 0.76
nucleotide binding 0.76
nucleoside phosphate binding 0.76
hydrolase activity, hydrolyzing N-glycosyl compounds 0.76
oxidoreductase activity, acting on the CH-OH group of donors, oxygen as acceptor 0.67
transferase activity, transferring glycosyl groups 0.64
poly-pyrimidine tract binding 0.57
thylakoid lumen 0.59
0
1
2
3
-log10fdr

MM4.skyblue

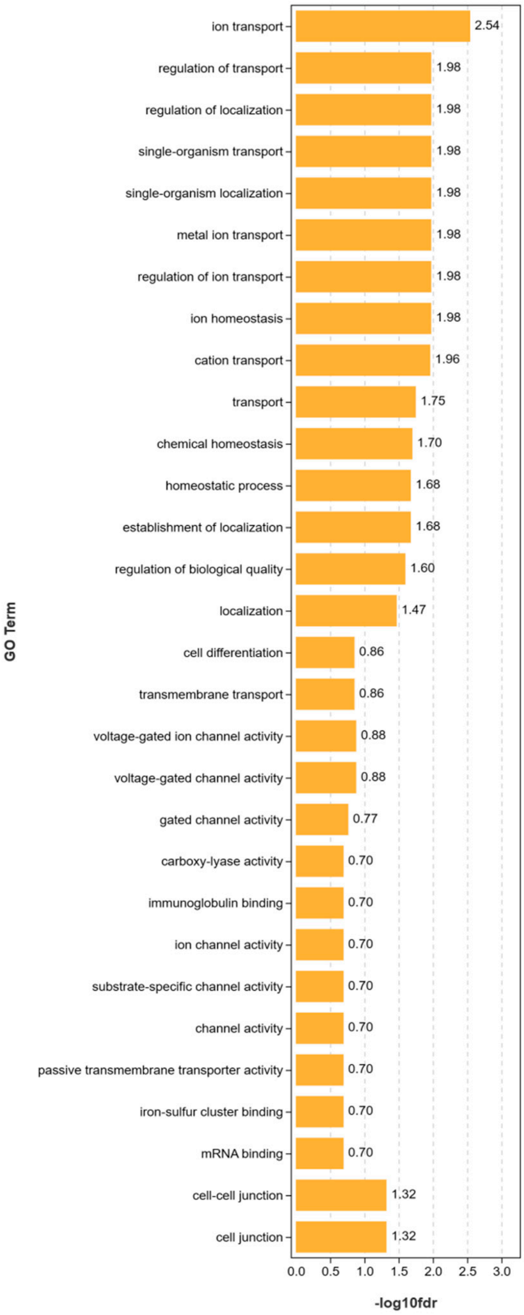

MM5.lightgreen

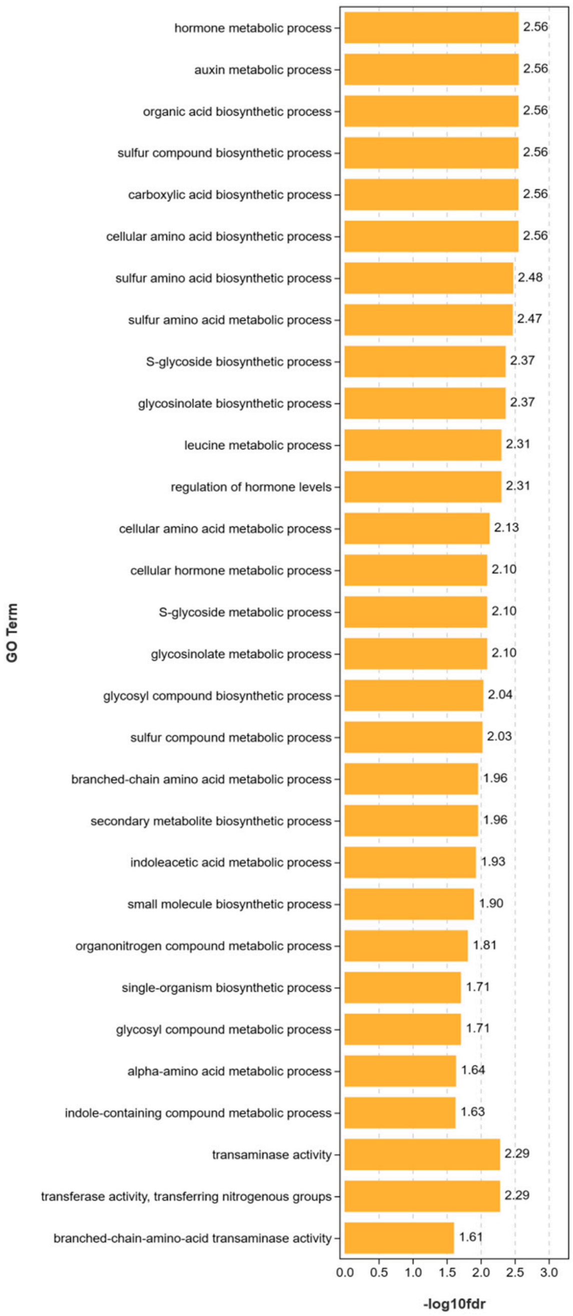

MM6.midnightblue

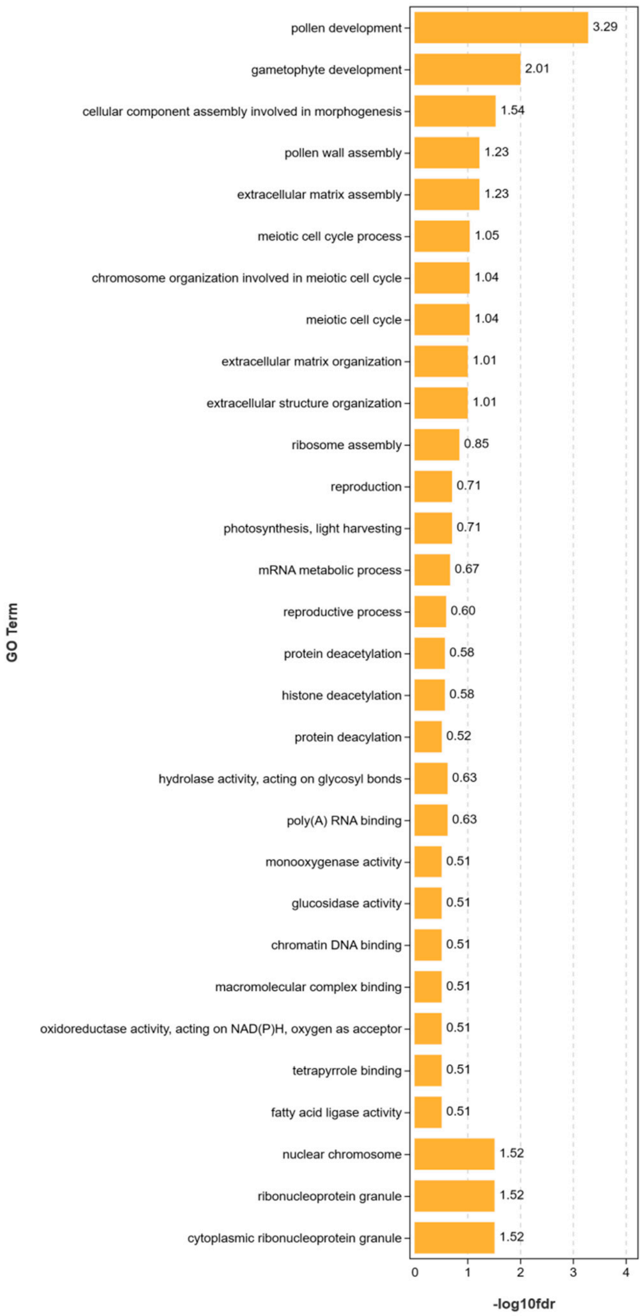

pollen development
gametophyte development
cellular component assembly involved in morphogenesis
pollen wall assembly
extracellular matrix assembly
meiotic cell cycle process
chromosome organization involved in meiotic cell cycle
meiotic cell cycle
extracellular matrix organization
extracellular structure organization
ribosome assembly
reproduction
photosynthesis, light harvesting
mRNA metabolic process
reproductive process
protein deacetylation
histone deacetylation
protein deacylation
hydrolase activity, acting on glycosyl bonds
poly(A) RNA binding
monooxygenase activity
glucosidase activity
chromatin DNA binding
macromolecular complex binding
oxidoreductase activity, acting on NAD(P)H, oxygen as acceptor
tetrapyrrole binding
fatty acid ligase activity
nuclear chromosome
ribonucleoprotein granule
cytoplasmic ribonucleoprotein granule
3.29
2.01
1.54
1.23
1.23
1.05
1.04
1.04
1.01
1.01
0.85
0.71
0.71
0.67
0.60
0.58
0.58
0.52
0.63
0.63
0.51
0.51
0.51
0.51
0.51
0.51
0.51
1.52
1.52
1.52
GO Term
0
1
2
3
4
-log10fdr

MM7.brown

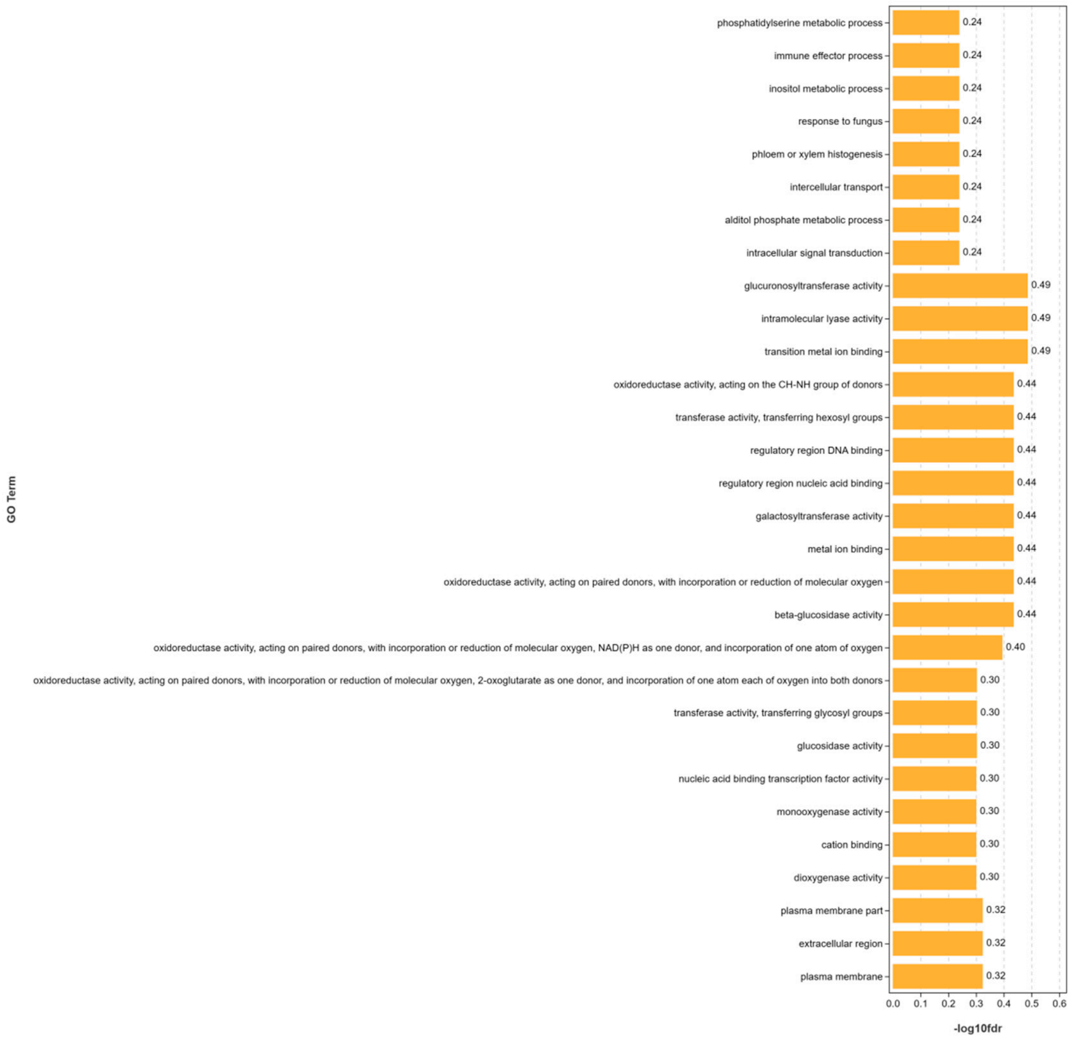
GO Term
phosphatidylserine metabolic process
immune effector process
inositol metabolic process
response to fungus
phloem or xylem histogenesis
intercellular transport
alditol phosphate metabolic process
intracellular signal transduction
glucuronosyltransferase activity
intramolecular lyase activity
transition metal ion binding
oxidoreductase activity, acting on the CH-NH group of donors
transferase activity, transferring hexosyl groups
regulatory region DNA binding
regulatory region nucleic acid binding
galactosyltransferase activity
metal ion binding
oxidoreductase activity, acting on paired donors, with incorporation or reduction of molecular oxygen
beta-glucosidase activity
oxidoreductase activity, acting on paired donors, with incorporation or reduction of molecular oxygen, NAD(P)H as one donor, and incorporation of one atom of oxygen
oxidoreductase activity, acting on paired donors, with incorporation or reduction of molecular oxygen, 2-oxoglutarate as one donor, and incorporation of one atom each of oxygen into both donors
transferase activity, transferring glycosyl groups
glucosidase activity
nucleic acid binding transcription factor activity
monooxygenase activity
cation binding
dioxygenase activity
plasma membrane part
extracellular region
plasma membrane
0.24
0.24
0.24
0.24
0.24
0.24
0.24
0.24
0.49
0.49
0.49
0.44
0.44
0.44
0.44
0.44
0.44
0.44
0.44
0.40
0.30
0.30
0.30
0.30
0.30
0.30
0.30
0.32
0.32
0.32
0.0 0.1 0.2 0.3 0.4 0.5 0.6
-log10fdr

MM8.orange

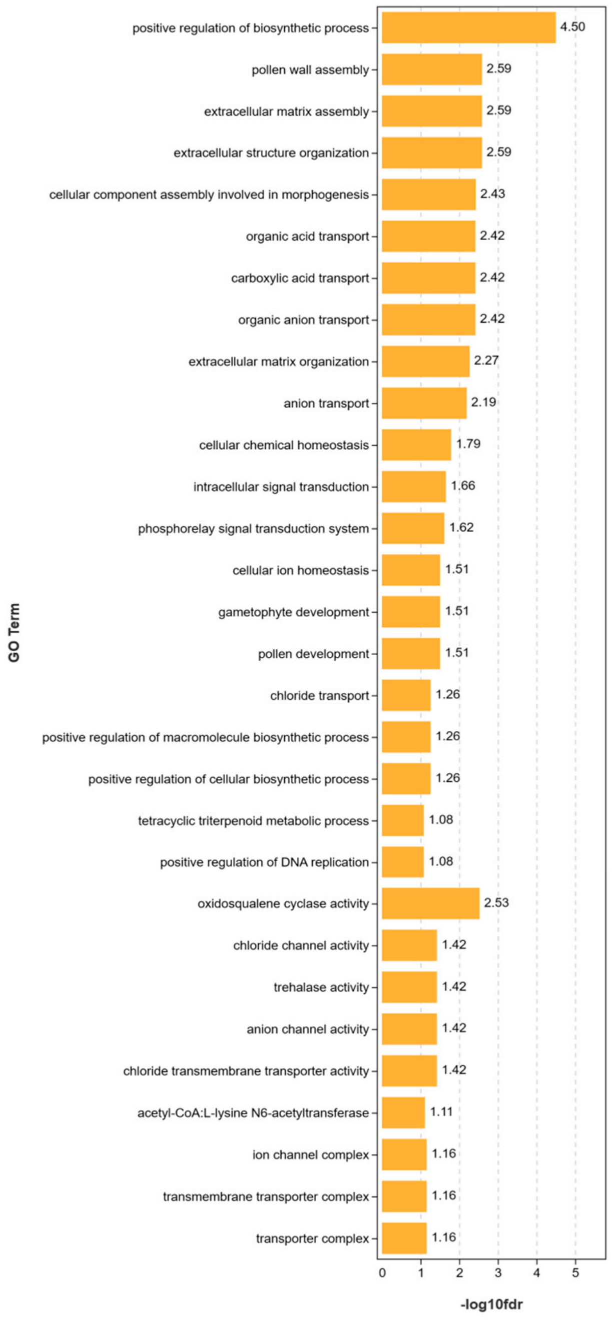

GO Term
positive regulation of biosynthetic process 4.50
pollen wall assembly 2.59
extracellular matrix assembly 2.59
extracellular structure organization 2.59
cellular component assembly involved in morphogenesis 2.43
organic acid transport 2.42
carboxylic acid transport 2.42
organic anion transport 2.42
extracellular matrix organization 2.27
anion transport 2.19
cellular chemical homeostasis 1.79
intracellular signal transduction 1.66
phosphorelay signal transduction system 1.62
cellular ion homeostasis 1.51
gametophyte development 1.51
pollen development 1.51
chloride transport 1.26
positive regulation of macromolecule biosynthetic process 1.26
positive regulation of cellular biosynthetic process 1.26
tetracyclic triterpenoid metabolic process 1.08
positive regulation of DNA replication 1.08
oxidosqualene cyclase activity 2.53
chloride channel activity 1.42
trehalase activity 1.42
anion channel activity 1.42
chloride transmembrane transporter activity 1.42
acetyl-CoA:L-lysine N6-acetyltransferase 1.11
ion channel complex 1.16
transmembrane transporter complex 1.16
transporter complex 1.16
0 1 2 3 4 5
-log10fdr

MM9.blue

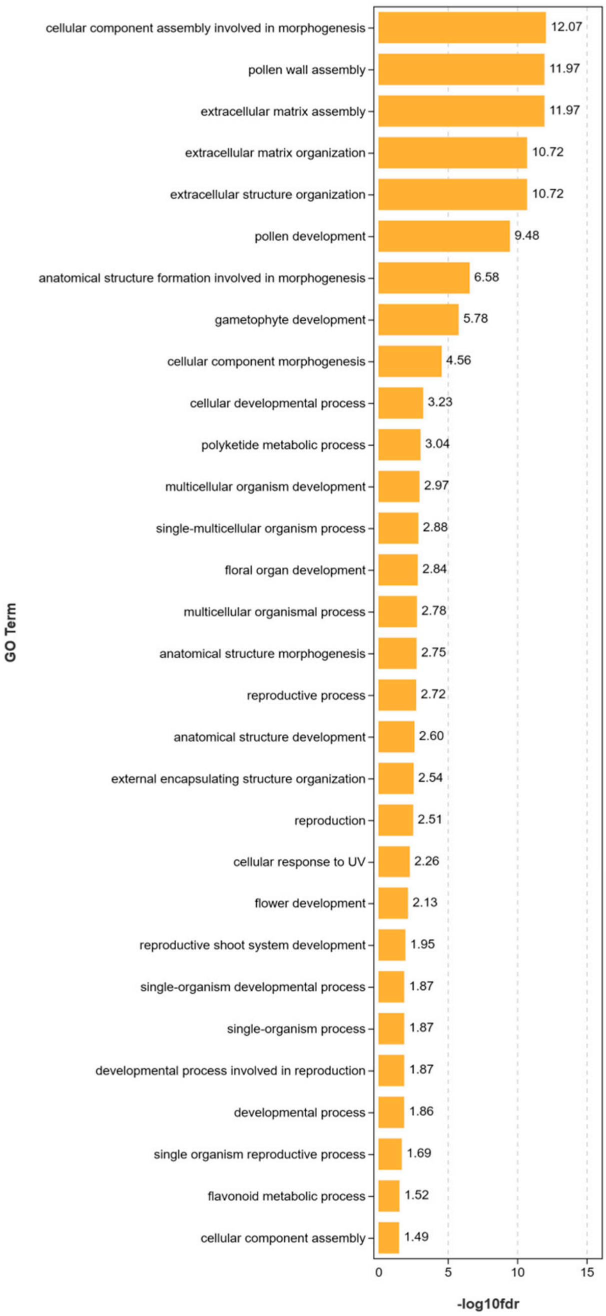
cellular component assembly involved in morphogenesis
12.07
pollen wall assembly
11.97
extracellular matrix assembly
11.97
extracellular matrix organization
10.72
extracellular structure organization
10.72
pollen development
9.48
anatomical structure formation involved in morphogenesis
6.58
gametophyte development
5.78
cellular component morphogenesis
4.56
cellular developmental process
3.23
polyketide metabolic process
3.04
multicellular organism development
2.97
single-multicellular organism process
2.88
floral organ development
2.84
multicellular organismal process
2.78
anatomical structure morphogenesis
2.75
reproductive process
2.72
anatomical structure development
2.60
external encapsulating structure organization
2.54
reproduction
2.51
cellular response to UV
2.26
flower development
2.13
reproductive shoot system development
1.95
single-organism developmental process
1.87
single-organism process
1.87
developmental process involved in reproduction
1.87
developmental process
1.86
single organism reproductive process
1.69
flavonoid metabolic process
1.52
cellular component assembly
1.49
GO Term
0
5
10
15
-log10fdr

MM10.pink

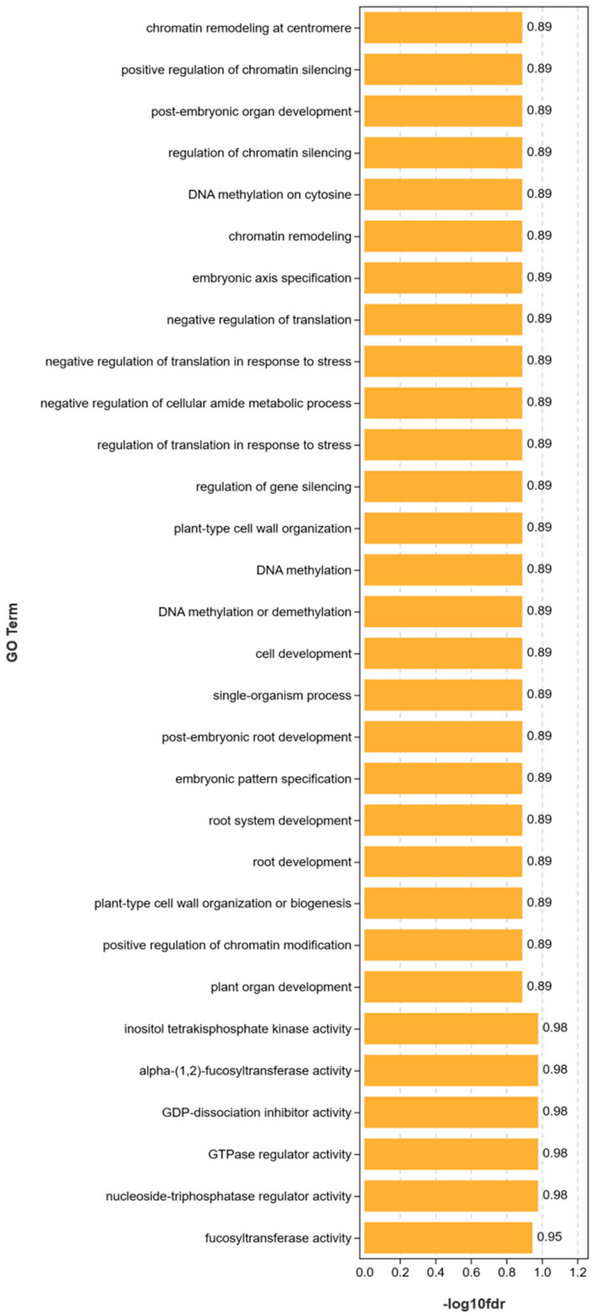

MM11.darkorange

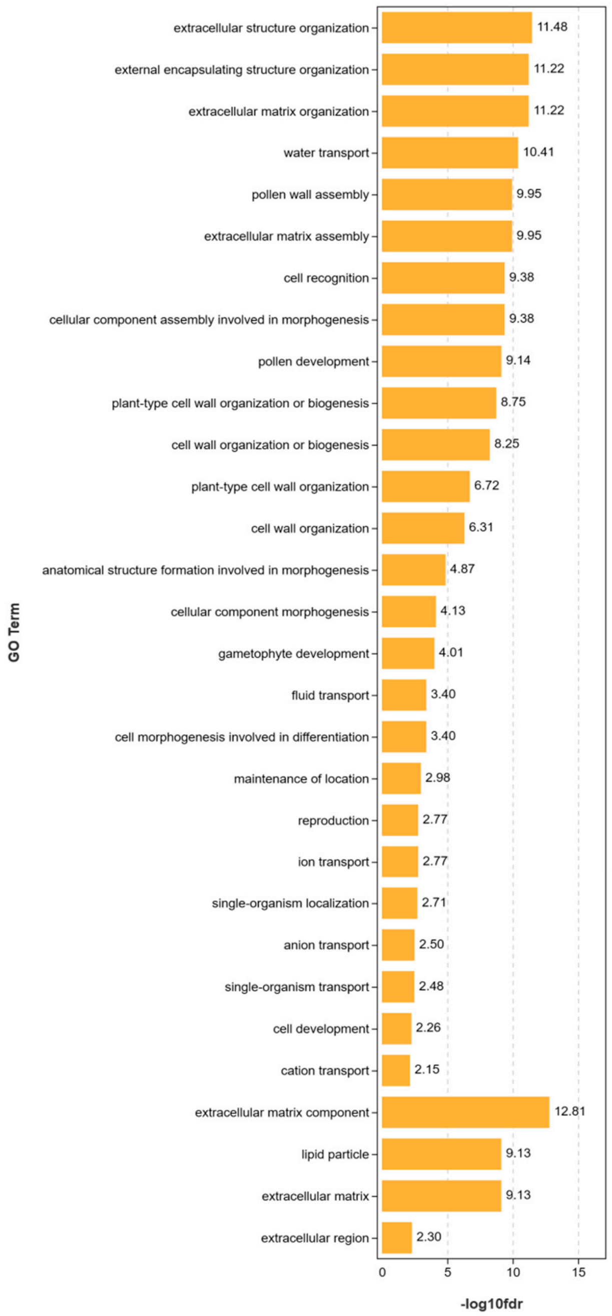

MM12.turquoise

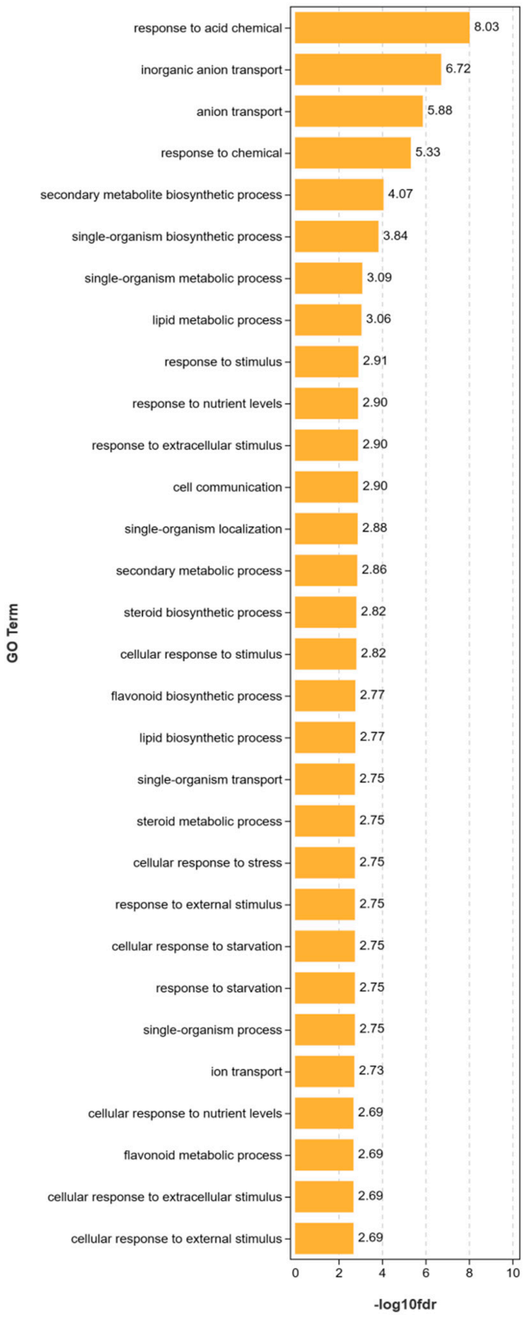
GO Term
response to acid chemical
8.03
inorganic anion transport
6.72
anion transport
5.88
response to chemical
5.33
secondary metabolite biosynthetic process
4.07
single-organism biosynthetic process
3.84
single-organism metabolic process
3.09
lipid metabolic process
3.06
response to stimulus
2.91
response to nutrient levels
2.90
response to extracellular stimulus
2.90
cell communication
2.90
single-organism localization
2.88
secondary metabolic process
2.86
steroid biosynthetic process
2.82
cellular response to stimulus
2.82
flavonoid biosynthetic process
2.77
lipid biosynthetic process
2.77
single-organism transport
2.75
steroid metabolic process
2.75
cellular response to stress
2.75
response to external stimulus
2.75
cellular response to starvation
2.75
response to starvation
2.75
single-organism process
2.75
ion transport
2.73
cellular response to nutrient levels
2.69
flavonoid metabolic process
2.69
cellular response to extracellular stimulus
2.69
cellular response to external stimulus
2.69
0
2
4
6
8
10
-log10fdr

MM13.cyan

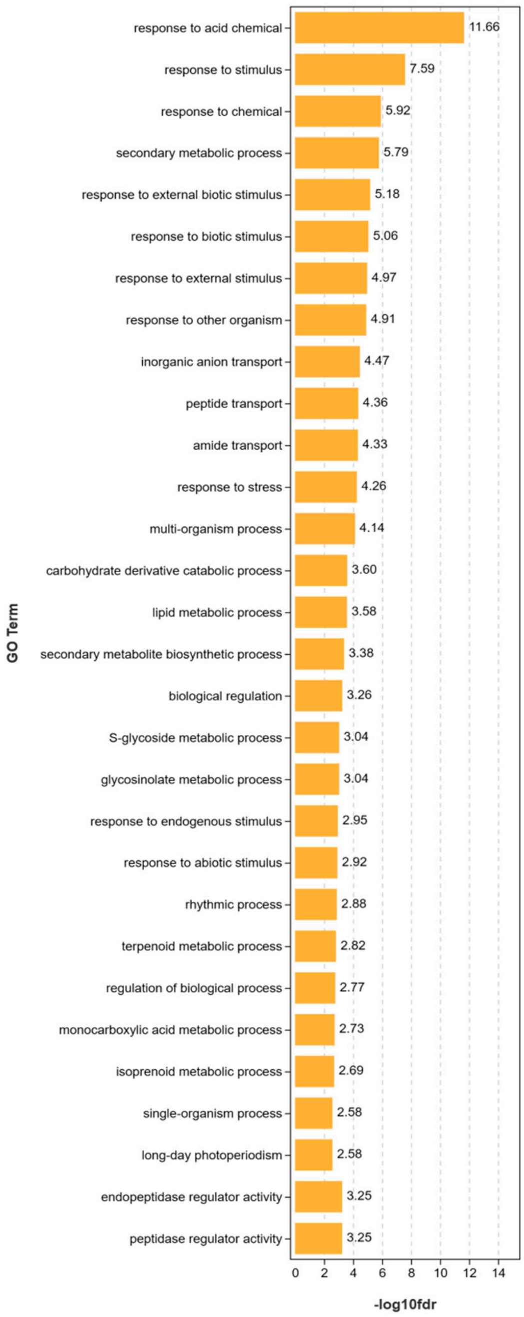

response to acid chemical
11.66
response to stimulus
7.59
response to chemical
5.92
secondary metabolic process
5.79
response to external biotic stimulus
5.18
response to biotic stimulus
5.06
response to external stimulus
4.97
response to other organism
4.91
inorganic anion transport
4.47
peptide transport
4.36
amide transport
4.33
response to stress
4.26
multi-organism process
4.14
carbohydrate derivative catabolic process
3.60
lipid metabolic process
3.58
secondary metabolite biosynthetic process
3.38
biological regulation
3.26
S-glycoside metabolic process
3.04
glycosinolate metabolic process
3.04
response to endogenous stimulus
2.95
response to abiotic stimulus
2.92
rhythmic process
2.88
terpenoid metabolic process
2.82
regulation of biological process
2.77
monocarboxylic acid metabolic process
2.73
isoprenoid metabolic process
2.69
single-organism process
2.58
long-day photoperiodism
2.58
endopeptidase regulator activity
3.25
peptidase regulator activity
3.25
GO Term
0 2 4 6 8 10 12 14
-log10fdr

MM14.tan

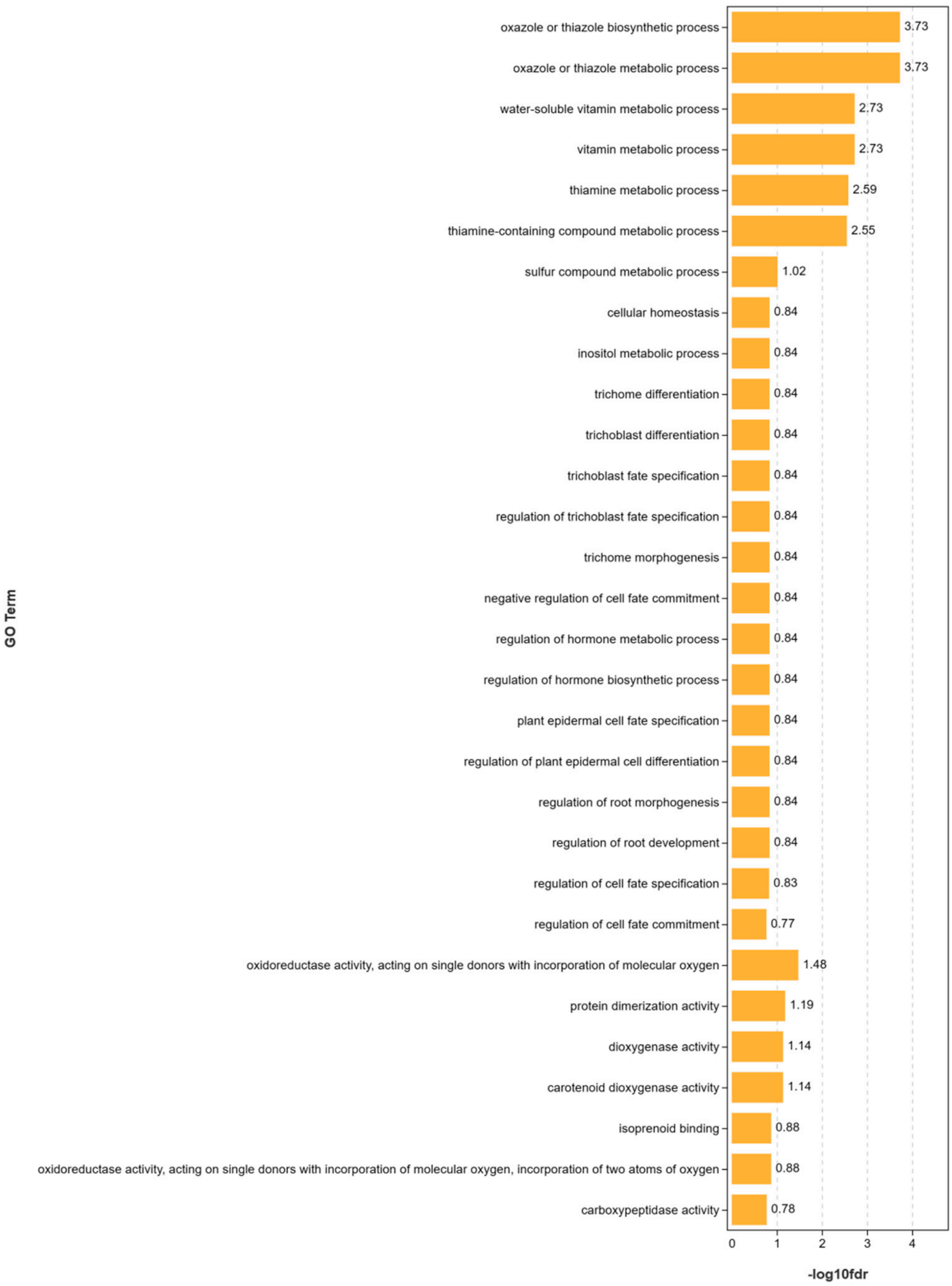

MM15.darkgrey

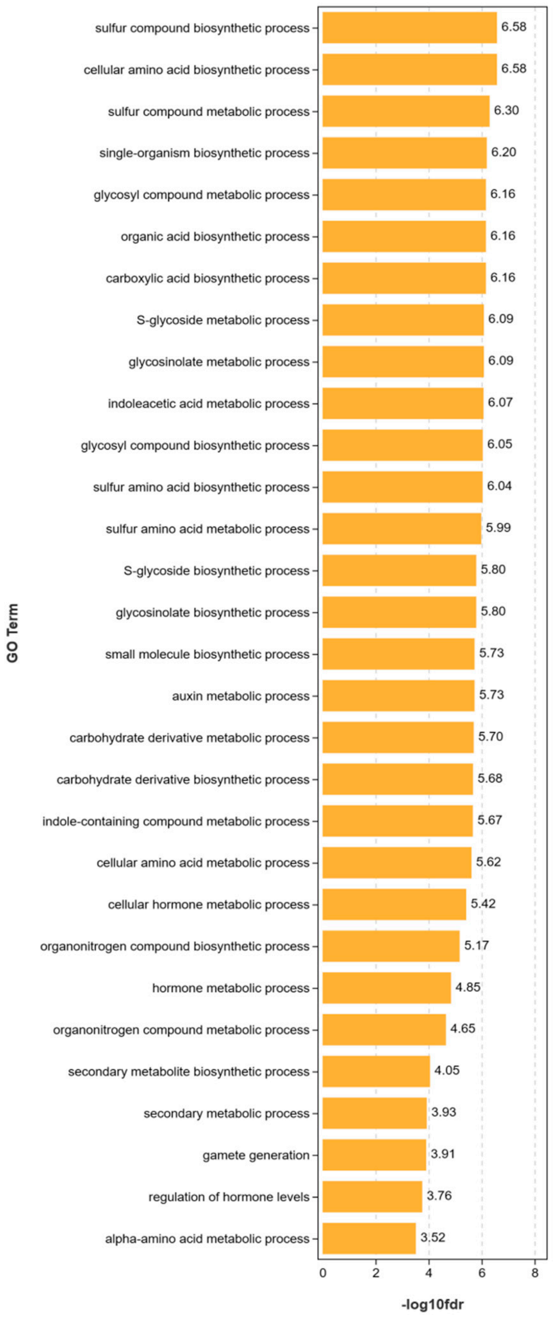

MM16.royalblue

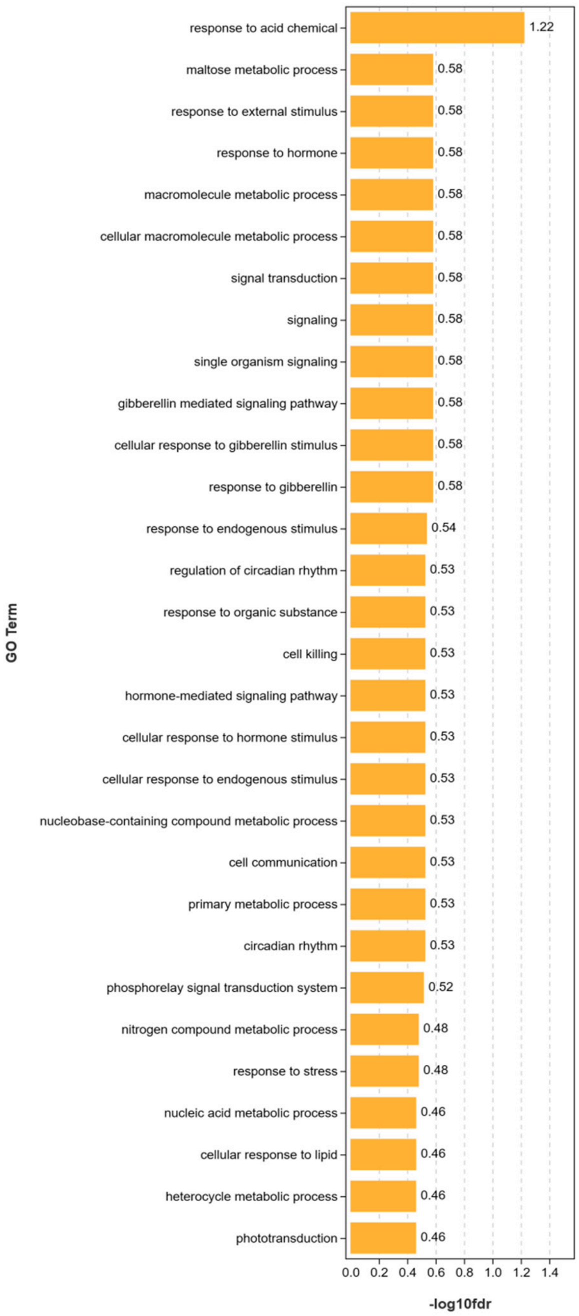

MM17.saddlebrown

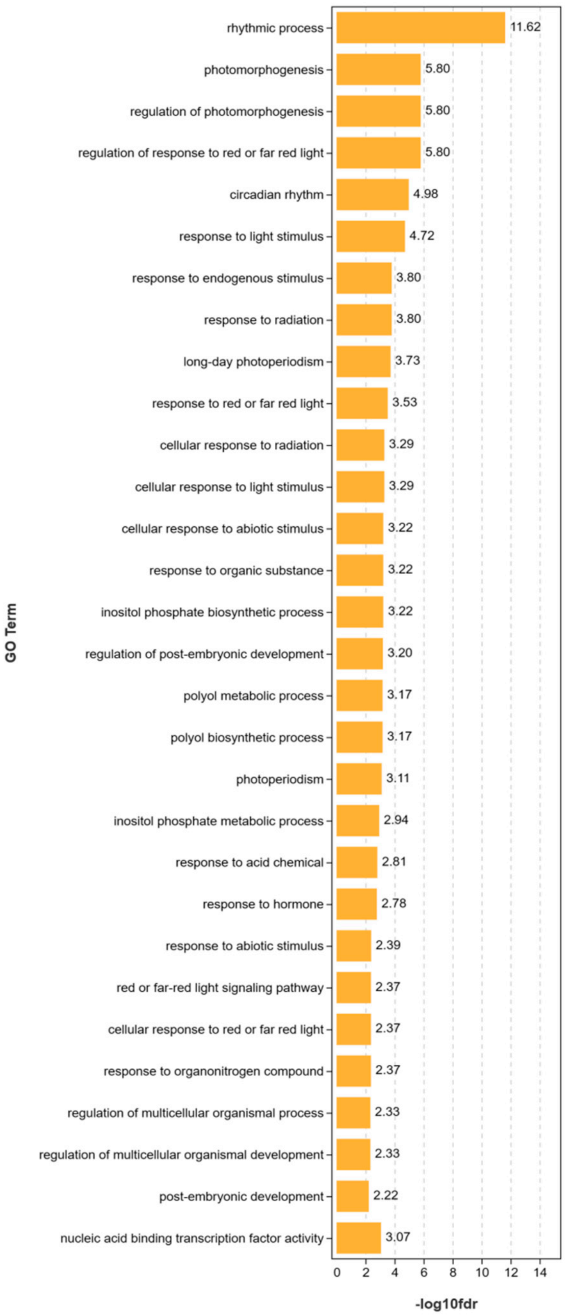
rhythmic process 11.62
photomorphogenesis 5.80
regulation of photomorphogenesis 5.80
regulation of response to red or far red light 5.80
circadian rhythm 4.98
response to light stimulus 4.72
response to endogenous stimulus 3.80
response to radiation 3.80
long-day photoperiodism 3.73
response to red or far red light 3.53
cellular response to radiation 3.29
cellular response to light stimulus 3.29
cellular response to abiotic stimulus 3.22
response to organic substance 3.22
inositol phosphate biosynthetic process 3.22
regulation of post-embryonic development 3.20
polyol metabolic process 3.17
polyol biosynthetic process 3.17
photoperiodism 3.11
inositol phosphate metabolic process 2.94
response to acid chemical 2.81
response to hormone 2.78
response to abiotic stimulus 2.39
red or far-red light signaling pathway 2.37
cellular response to red or far red light 2.37
response to organonitrogen compound 2.37
regulation of multicellular organismal process 2.33
regulation of multicellular organismal development 2.33
post-embryonic development 2.22
nucleic acid binding transcription factor activity 3.07
GO Term
0 2 4 6 8 10 12 14
-log10fdr

MM18.salmon
