# Supplementary material for: Transcriptome Analysis of Intermittent Light Induced Early Bolting in Flowering Chinese Cabbage
Source: Plants (Basel). 2024 Mar 17;13(6):866. doi: 10.3390/plants13060866 (PMC10975546; doi:10.3390/plants13060866)
Supplement: Supplementary file 1 [file plants-13-00866-s001.zip › Figure S5.pdf]

Figure S5. KEGG Concentration Map of DEGs Between 18 Modules

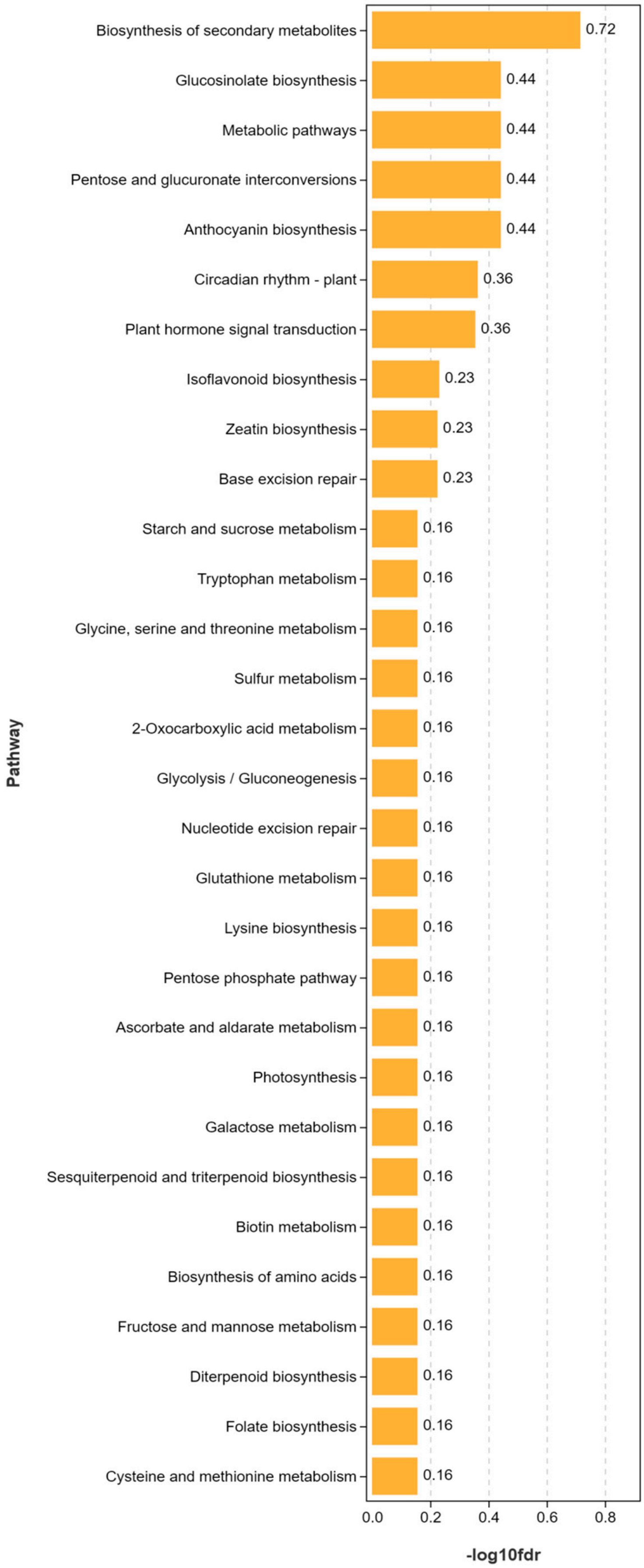

Biosynthesis of secondary metabolites
Glucosinolate biosynthesis
Metabolic pathways
Pentose and glucuronate interconversions
Anthocyanin biosynthesis
Circadian rhythm - plant
Plant hormone signal transduction
Isoflavonoid biosynthesis
Zeatin biosynthesis
Base excision repair
Starch and sucrose metabolism
Tryptophan metabolism
Glycine, serine and threonine metabolism
Sulfur metabolism
2-Oxocarboxylic acid metabolism
Glycolysis / Gluconeogenesis
Nucleotide excision repair
Glutathione metabolism
Lysine biosynthesis
Pentose phosphate pathway
Ascorbate and aldarate metabolism
Photosynthesis
Galactose metabolism
Sesquiterpenoid and triterpenoid biosynthesis
Biotin metabolism
Biosynthesis of amino acids
Fructose and mannose metabolism
Diterpenoid biosynthesis
Folate biosynthesis
Cysteine and methionine metabolism
0.72
0.44
0.44
0.44
0.44
0.36
0.36
0.23
0.23
0.23
0.16
Pathway
0.0
0.2
0.4
0.6
0.8
-log10fdr

MM1.green

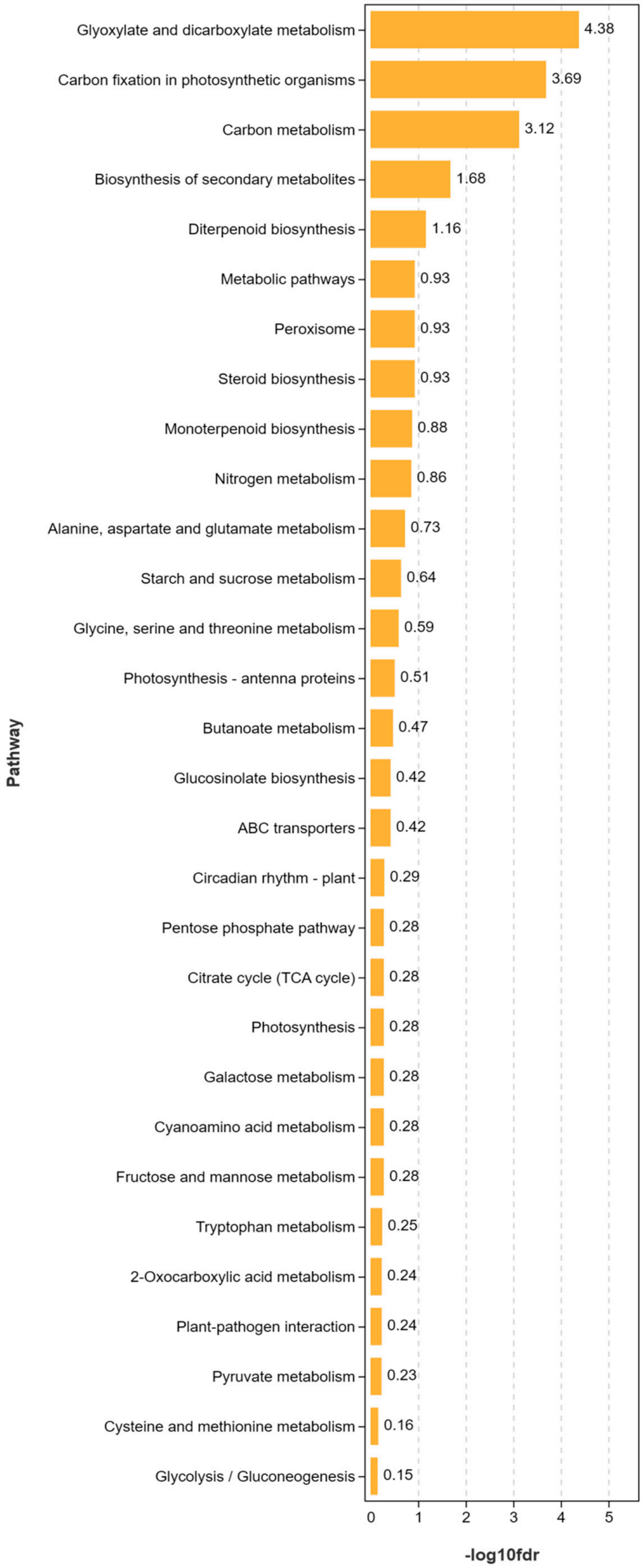

Glyoxylate and dicarboxylate metabolism
Carbon fixation in photosynthetic organisms
Carbon metabolism
Biosynthesis of secondary metabolites
Diterpenoid biosynthesis
Metabolic pathways
Peroxisome
Steroid biosynthesis
Monoterpenoid biosynthesis
Nitrogen metabolism
Alanine, aspartate and glutamate metabolism
Starch and sucrose metabolism
Glycine, serine and threonine metabolism
Photosynthesis - antenna proteins
Butanoate metabolism
Glucosinolate biosynthesis
ABC transporters
Circadian rhythm - plant
Pentose phosphate pathway
Citrate cycle (TCA cycle)
Photosynthesis
Galactose metabolism
Cyanoamino acid metabolism
Fructose and mannose metabolism
Tryptophan metabolism
2-Oxocarboxylic acid metabolism
Plant-pathogen interaction
Pyruvate metabolism
Cysteine and methionine metabolism
Glycolysis / Gluconeogenesis
4.38
3.69
3.12
1.68
1.16
0.93
0.93
0.93
0.88
0.86
0.73
0.64
0.59
0.51
0.47
0.42
0.42
0.29
0.28
0.28
0.28
0.28
0.28
0.28
0.25
0.24
0.24
0.23
0.16
0.15
Pathway
0
1
2
3
4
5
-log10fdr

MM2.purple

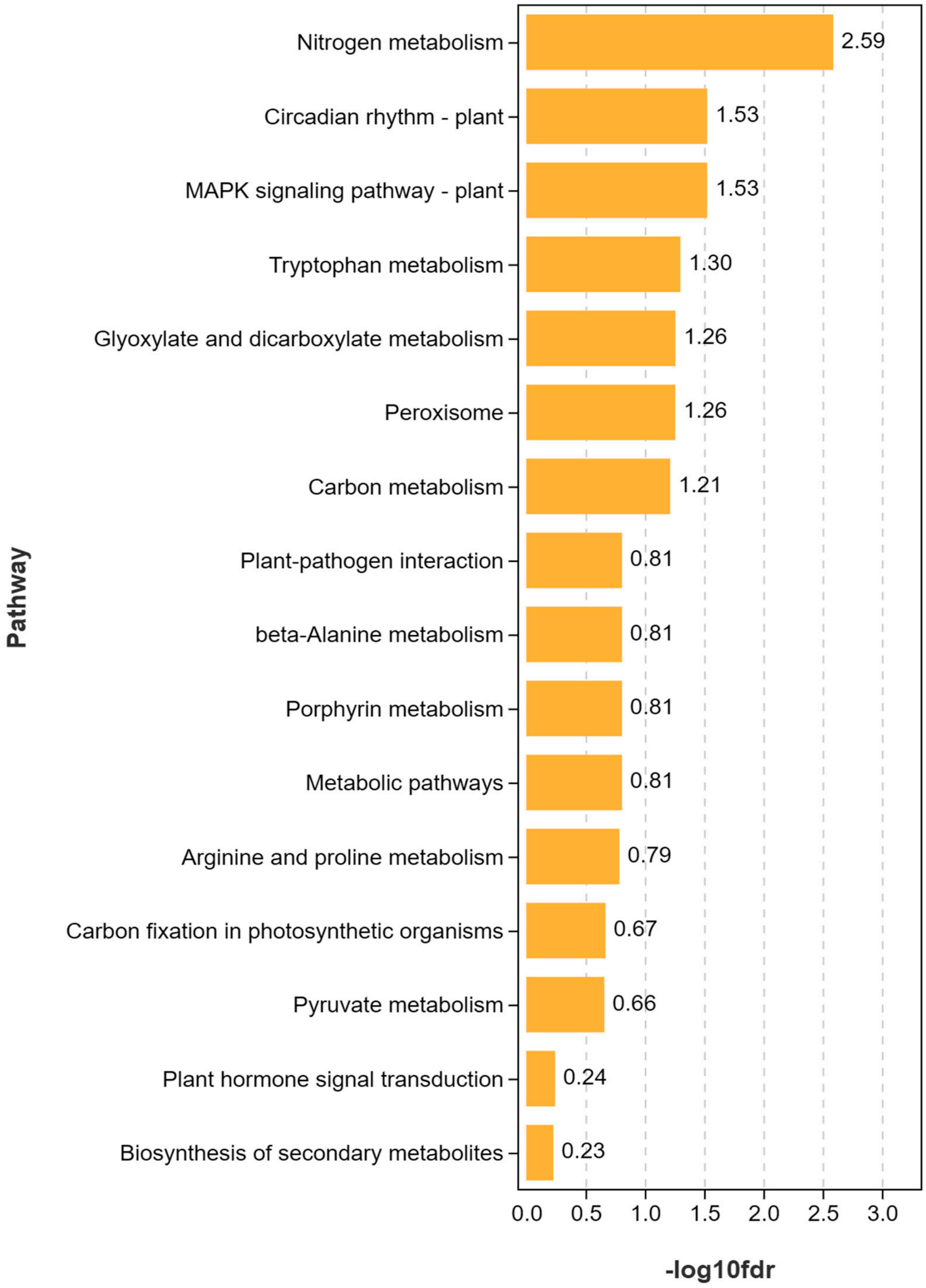

MM3.darkred

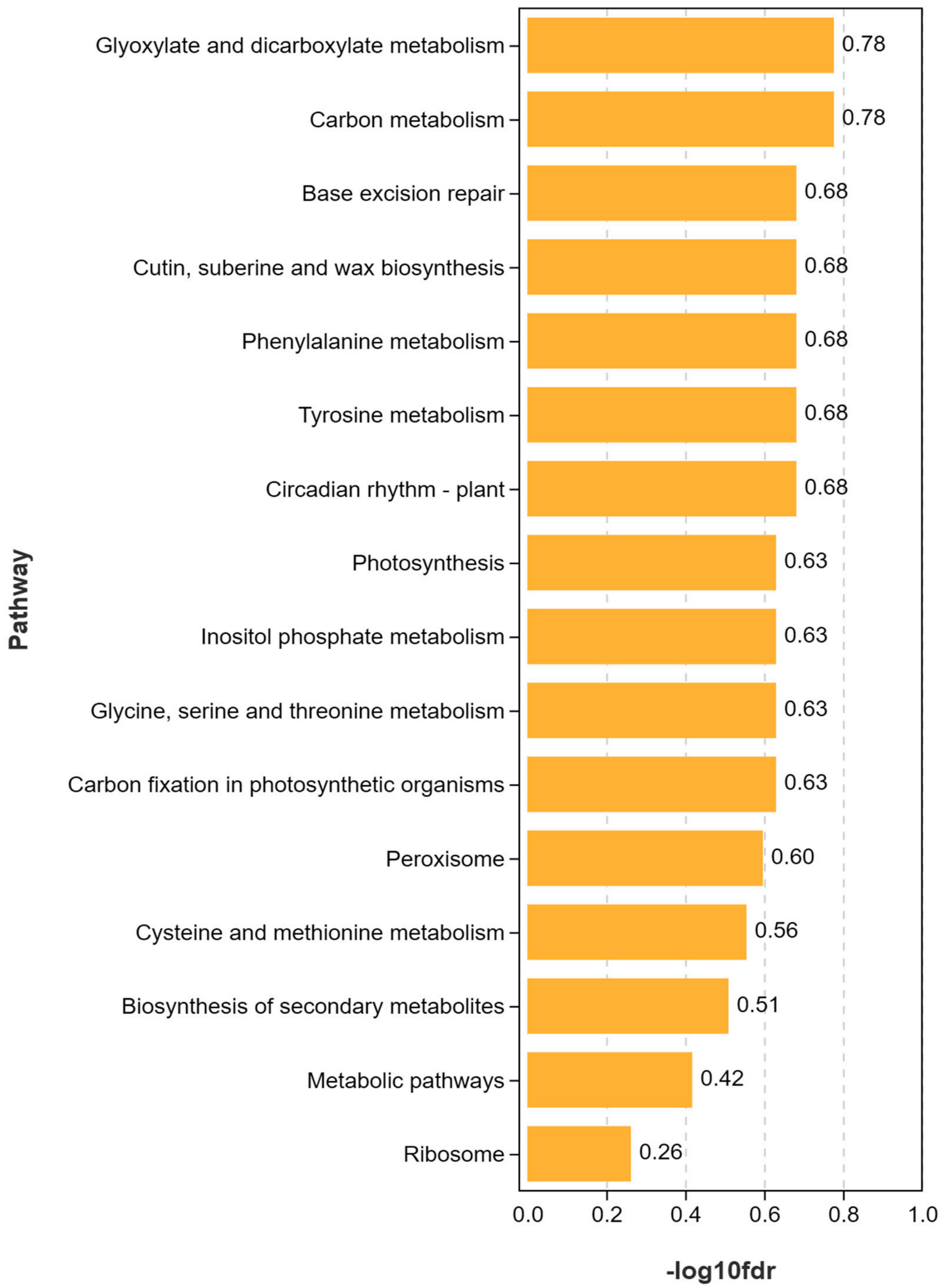
Glyoxylate and dicarboxylate metabolism
Carbon metabolism
Base excision repair
Cutin, suberine and wax biosynthesis
Phenylalanine metabolism
Tyrosine metabolism
Circadian rhythm - plant
Photosynthesis
Inositol phosphate metabolism
Glycine, serine and threonine metabolism
Carbon fixation in photosynthetic organisms
Peroxisome
Cysteine and methionine metabolism
Biosynthesis of secondary metabolites
Metabolic pathways
Ribosome
0.78
0.78
0.68
0.68
0.68
0.68
0.68
0.63
0.63
0.63
0.63
0.60
0.56
0.51
0.42
0.26
Pathway
0.0
0.2
0.4
0.6
0.8
1.0
-log10fdr

MM4.skyblue

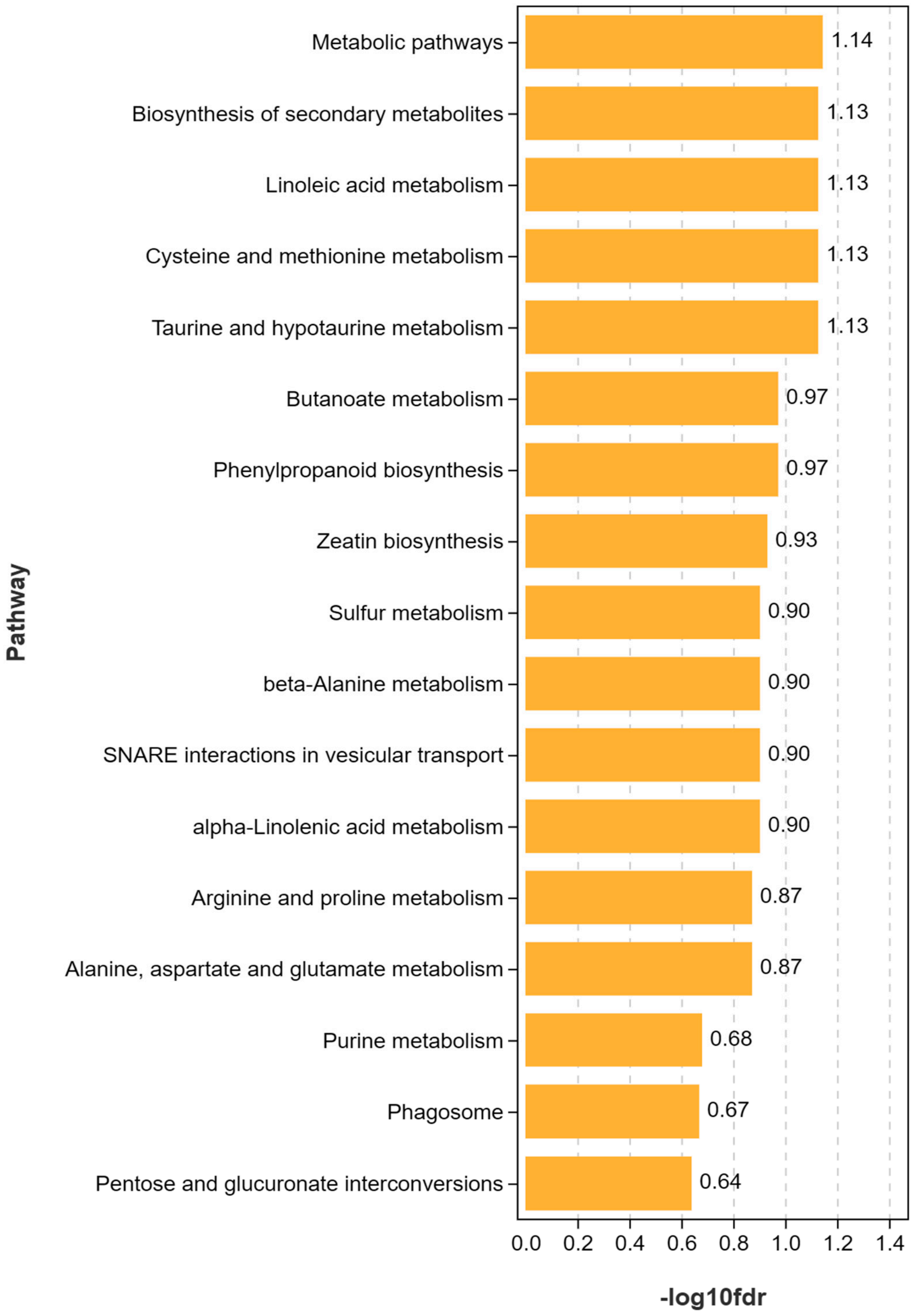

MM5.lightgreen

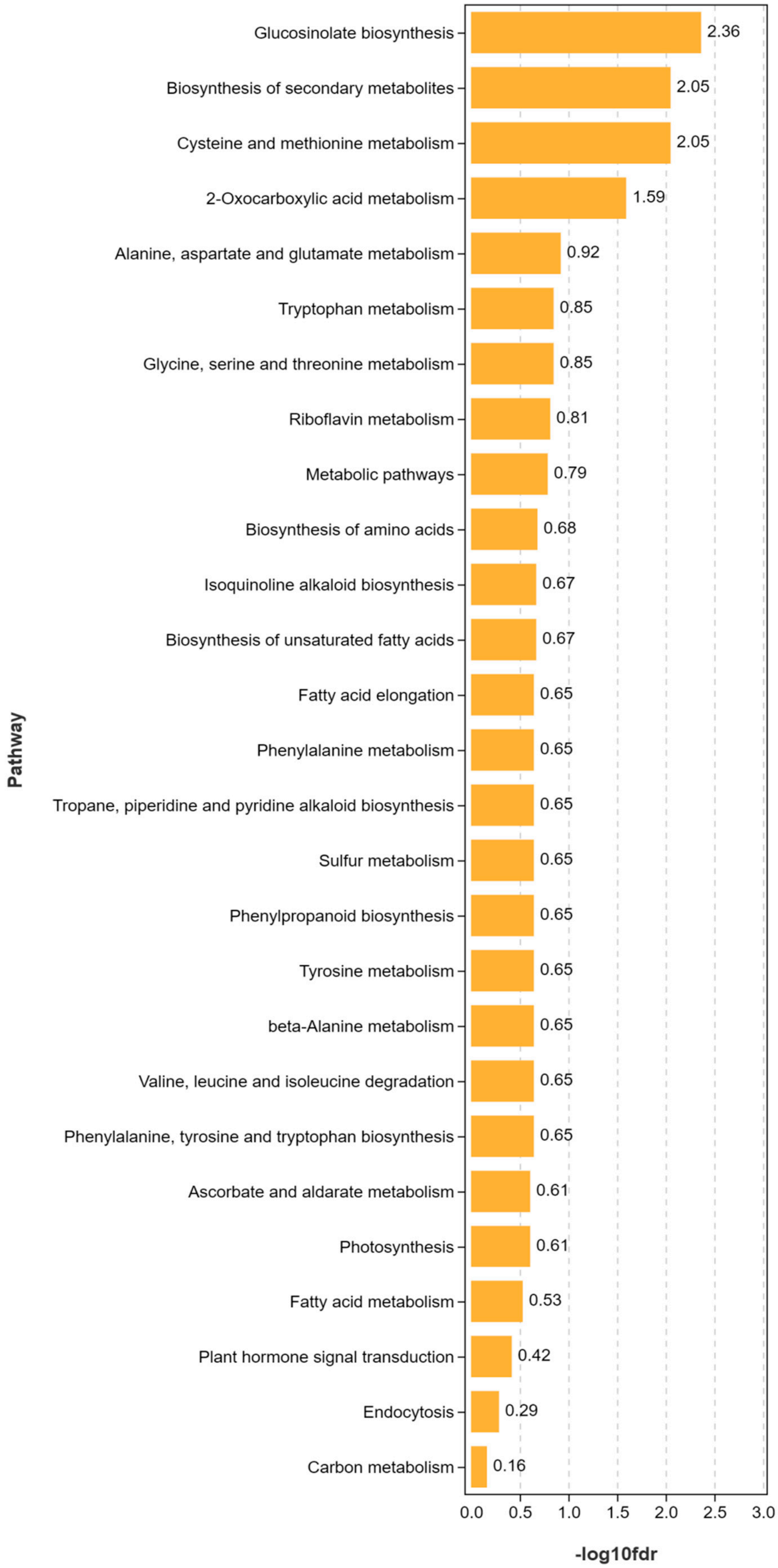
Pathway
-log10fdr
Glucosinolate biosynthesis
Biosynthesis of secondary metabolites
Cysteine and methionine metabolism
2-Oxocarboxylic acid metabolism
Alanine, aspartate and glutamate metabolism
Tryptophan metabolism
Glycine, serine and threonine metabolism
Riboflavin metabolism
Metabolic pathways
Biosynthesis of amino acids
Isoquinoline alkaloid biosynthesis
Biosynthesis of unsaturated fatty acids
Fatty acid elongation
Phenylalanine metabolism
Tropane, piperidine and pyridine alkaloid biosynthesis
Sulfur metabolism
Phenylpropanoid biosynthesis
Tyrosine metabolism
beta-Alanine metabolism
Valine, leucine and isoleucine degradation
Phenylalanine, tyrosine and tryptophan biosynthesis
Ascorbate and aldarate metabolism
Photosynthesis
Fatty acid metabolism
Plant hormone signal transduction
Endocytosis
Carbon metabolism
2.36
2.05
2.05
1.59
0.92
0.85
0.85
0.81
0.79
0.68
0.67
0.67
0.65
0.65
0.65
0.65
0.65
0.65
0.65
0.65
0.65
0.61
0.61
0.53
0.42
0.29
0.16
0.0
0.5
1.0
1.5
2.0
2.5
3.0

MM6.midnightblue

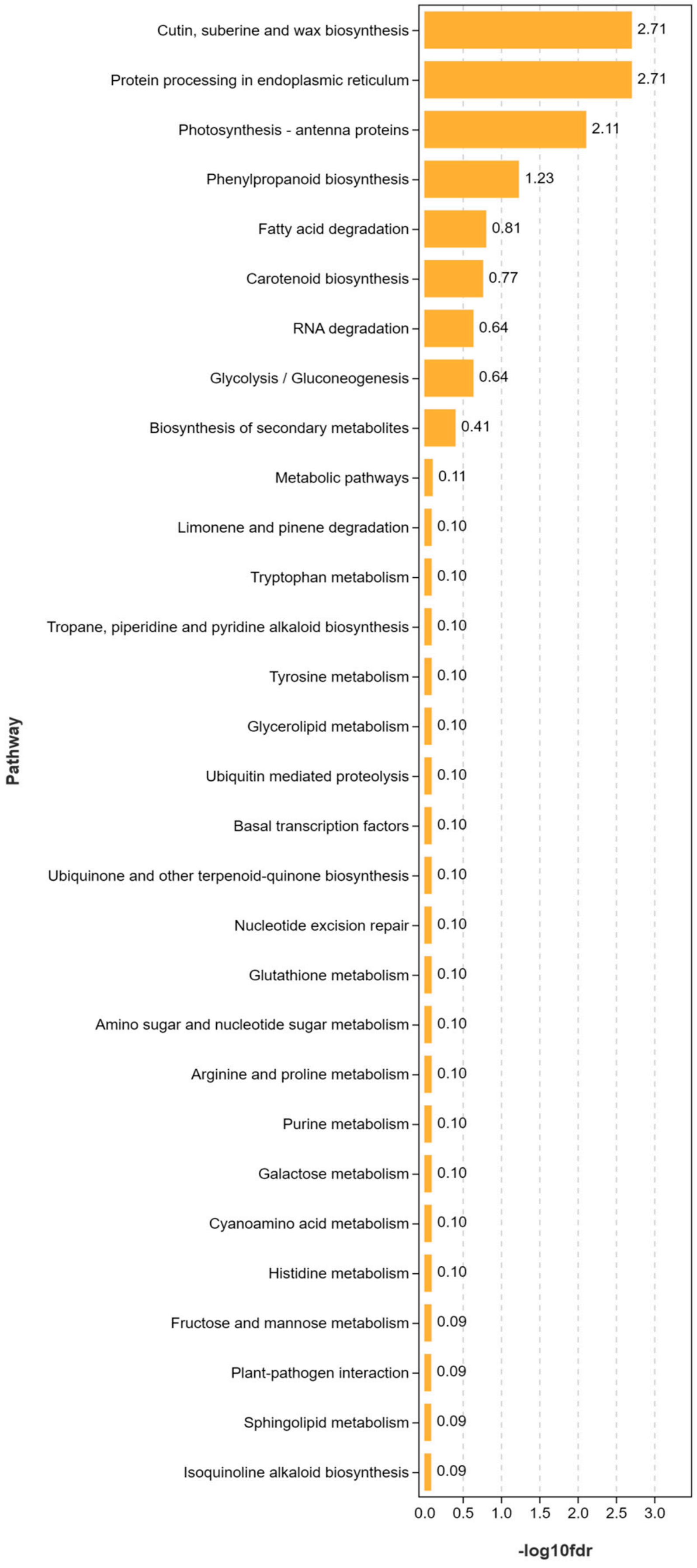
Cutin, suberine and wax biosynthesis
2.71
Protein processing in endoplasmic reticulum
2.71
Photosynthesis - antenna proteins
2.11
Phenylpropanoid biosynthesis
1.23
Fatty acid degradation
0.81
Carotenoid biosynthesis
0.77
RNA degradation
0.64
Glycolysis / Gluconeogenesis
0.64
Biosynthesis of secondary metabolites
0.41
Metabolic pathways
0.11
Limonene and pinene degradation
0.10
Tryptophan metabolism
0.10
Tropane, piperidine and pyridine alkaloid biosynthesis
0.10
Tyrosine metabolism
0.10
Glycerolipid metabolism
0.10
Ubiquitin mediated proteolysis
0.10
Basal transcription factors
0.10
Ubiquinone and other terpenoid-quinone biosynthesis
0.10
Nucleotide excision repair
0.10
Glutathione metabolism
0.10
Amino sugar and nucleotide sugar metabolism
0.10
Arginine and proline metabolism
0.10
Purine metabolism
0.10
Galactose metabolism
0.10
Cyanoamino acid metabolism
0.10
Histidine metabolism
0.10
Fructose and mannose metabolism
0.09
Plant-pathogen interaction
0.09
Sphingolipid metabolism
0.09
Isoquinoline alkaloid biosynthesis
0.09
Pathway
0.0 0.5 1.0 1.5 2.0 2.5 3.0
-log10fdr

MM7.brown

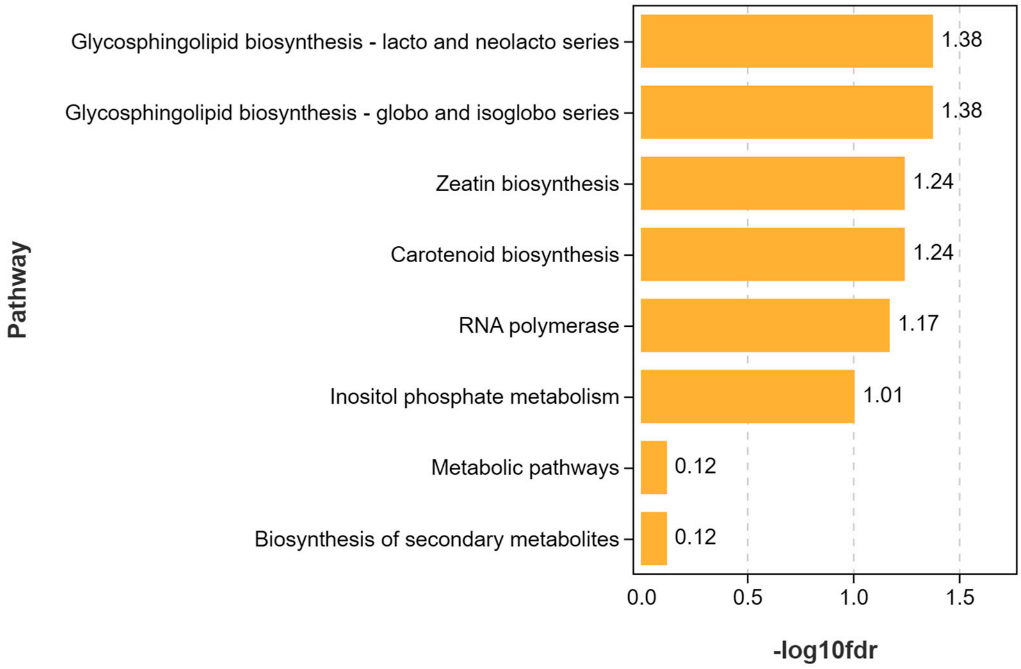

MM8.orange

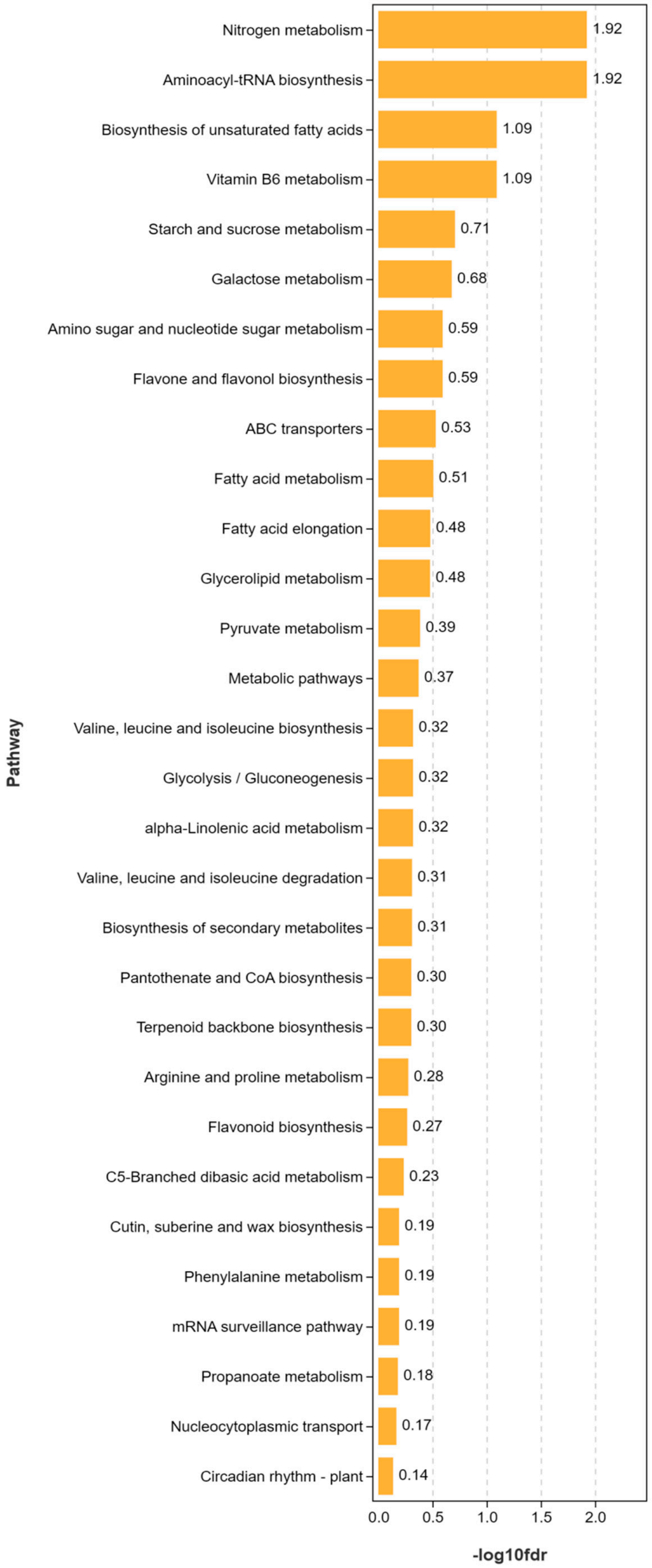

Pathway
Nitrogen metabolism
Aminoacyl-tRNA biosynthesis
Biosynthesis of unsaturated fatty acids
Vitamin B6 metabolism
Starch and sucrose metabolism
Galactose metabolism
Amino sugar and nucleotide sugar metabolism
Flavone and flavonol biosynthesis
ABC transporters
Fatty acid metabolism
Fatty acid elongation
Glycerolipid metabolism
Pyruvate metabolism
Metabolic pathways
Valine, leucine and isoleucine biosynthesis
Glycolysis / Gluconeogenesis
alpha-Linolenic acid metabolism
Valine, leucine and isoleucine degradation
Biosynthesis of secondary metabolites
Pantothenate and CoA biosynthesis
Terpenoid backbone biosynthesis
Arginine and proline metabolism
Flavonoid biosynthesis
C5-Branched dibasic acid metabolism
Cutin, suberine and wax biosynthesis
Phenylalanine metabolism
mRNA surveillance pathway
Propanoate metabolism
Nucleocytoplasmic transport
Circadian rhythm - plant
1.92
1.92
1.09
1.09
0.71
0.68
0.59
0.59
0.53
0.51
0.48
0.48
0.39
0.37
0.32
0.32
0.32
0.31
0.31
0.30
0.30
0.28
0.27
0.23
0.19
0.19
0.19
0.18
0.17
0.14
0.0
0.5
1.0
1.5
2.0
-log10fdr

MM9.blue

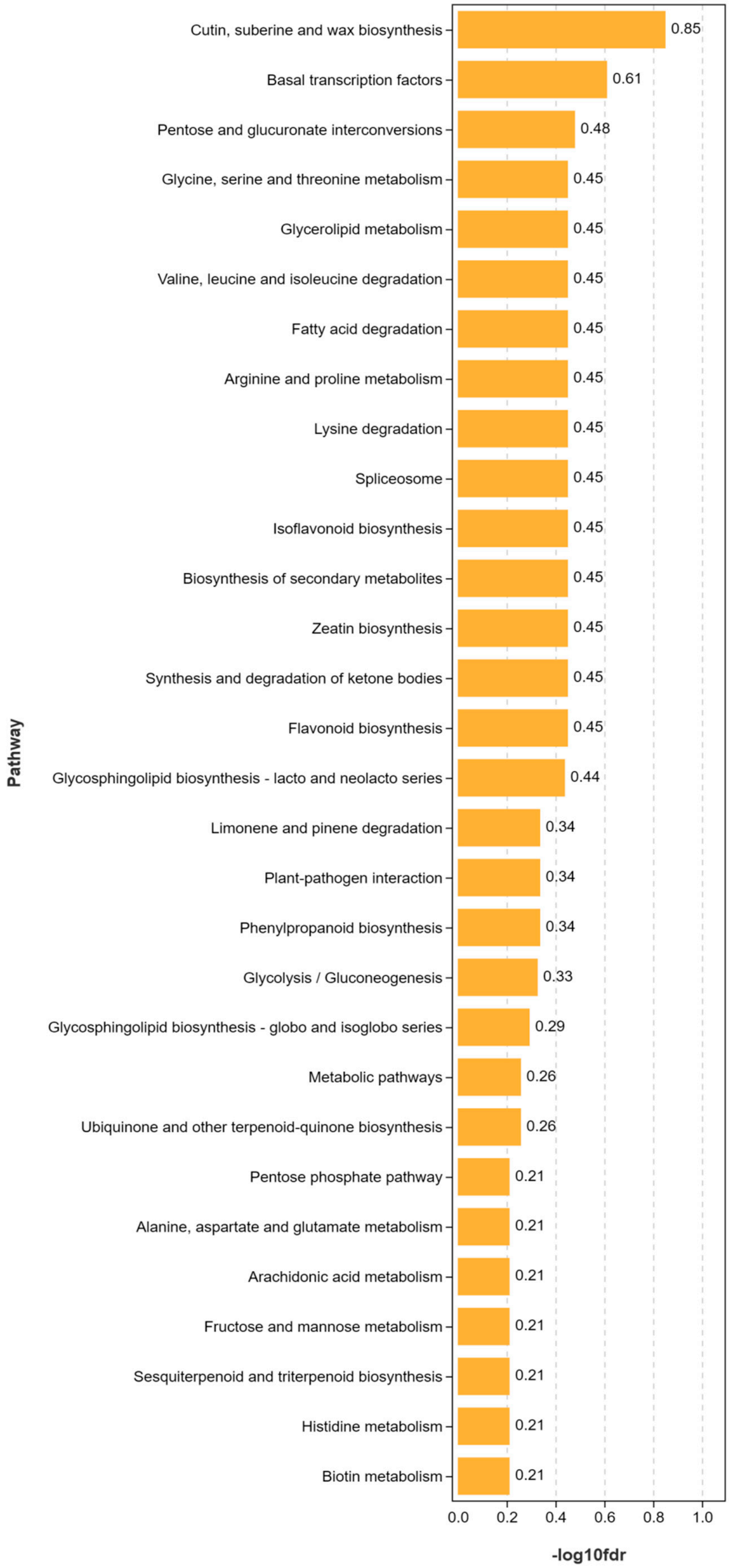
Cutin, suberine and wax biosynthesis
0.85
Basal transcription factors
0.61
Pentose and glucuronate interconversions
0.48
Glycine, serine and threonine metabolism
0.45
Glycerolipid metabolism
0.45
Valine, leucine and isoleucine degradation
0.45
Fatty acid degradation
0.45
Arginine and proline metabolism
0.45
Lysine degradation
0.45
Spliceosome
0.45
Isoflavonoid biosynthesis
0.45
Biosynthesis of secondary metabolites
0.45
Zeatin biosynthesis
0.45
Synthesis and degradation of ketone bodies
0.45
Flavonoid biosynthesis
0.45
Glycosphingolipid biosynthesis - lacto and neolacto series
0.44
Limonene and pinene degradation
0.34
Plant-pathogen interaction
0.34
Phenylpropanoid biosynthesis
0.34
Glycolysis / Gluconeogenesis
0.33
Glycosphingolipid biosynthesis - globo and isoglobo series
0.29
Metabolic pathways
0.26
Ubiquinone and other terpenoid-quinone biosynthesis
0.26
Pentose phosphate pathway
0.21
Alanine, aspartate and glutamate metabolism
0.21
Arachidonic acid metabolism
0.21
Fructose and mannose metabolism
0.21
Sesquiterpenoid and triterpenoid biosynthesis
0.21
Histidine metabolism
0.21
Biotin metabolism
0.21
Pathway
0.0
0.2
0.4
0.6
0.8
1.0
-log10fdr

MM10.pink

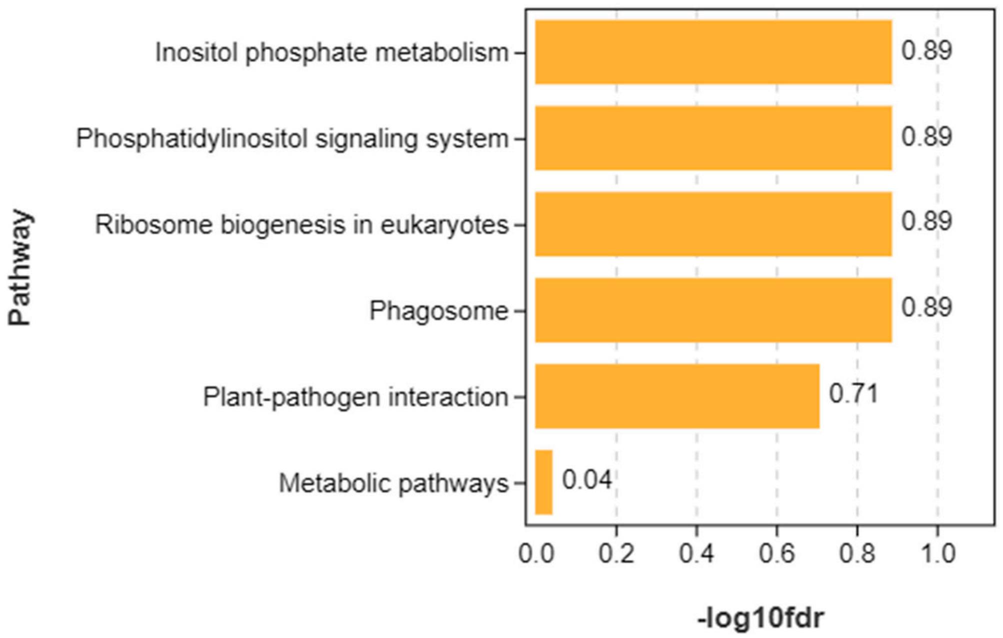

MM11.darkorange

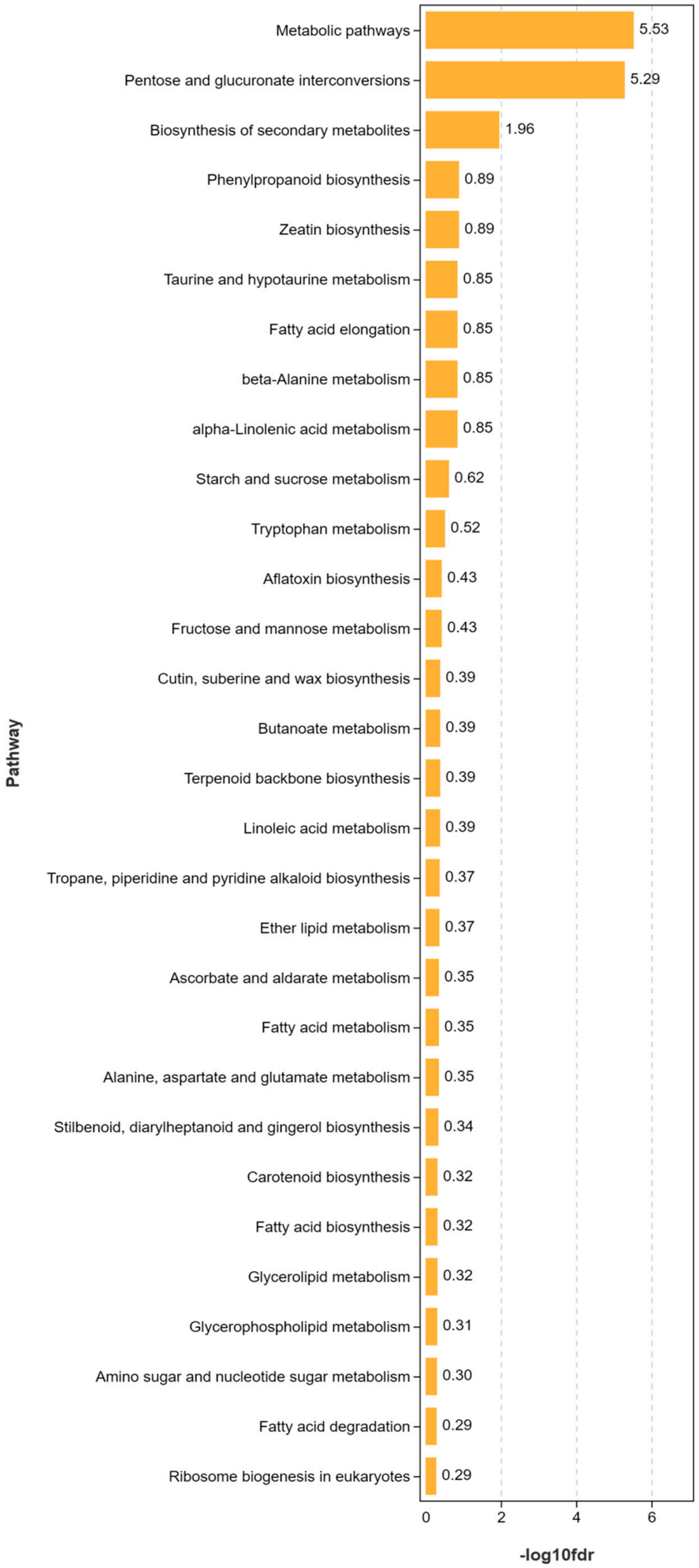

Pathway
Metabolic pathways
Pentose and glucuronate interconversions
Biosynthesis of secondary metabolites
Phenylpropanoid biosynthesis
Zeatin biosynthesis
Taurine and hypotaurine metabolism
Fatty acid elongation
beta-Alanine metabolism
alpha-Linolenic acid metabolism
Starch and sucrose metabolism
Tryptophan metabolism
Aflatoxin biosynthesis
Fructose and mannose metabolism
Cutin, suberine and wax biosynthesis
Butanoate metabolism
Terpenoid backbone biosynthesis
Linoleic acid metabolism
Tropane, piperidine and pyridine alkaloid biosynthesis
Ether lipid metabolism
Ascorbate and aldarate metabolism
Fatty acid metabolism
Alanine, aspartate and glutamate metabolism
Stilbenoid, diarylheptanoid and gingerol biosynthesis
Carotenoid biosynthesis
Fatty acid biosynthesis
Glycerolipid metabolism
Glycerophospholipid metabolism
Amino sugar and nucleotide sugar metabolism
Fatty acid degradation
Ribosome biogenesis in eukaryotes
5.53
5.29
1.96
0.89
0.89
0.85
0.85
0.85
0.85
0.62
0.52
0.43
0.43
0.39
0.39
0.39
0.39
0.37
0.37
0.35
0.35
0.35
0.34
0.32
0.32
0.32
0.31
0.30
0.29
0.29
0
2
4
6
-log10fdr

MM12.turquoise

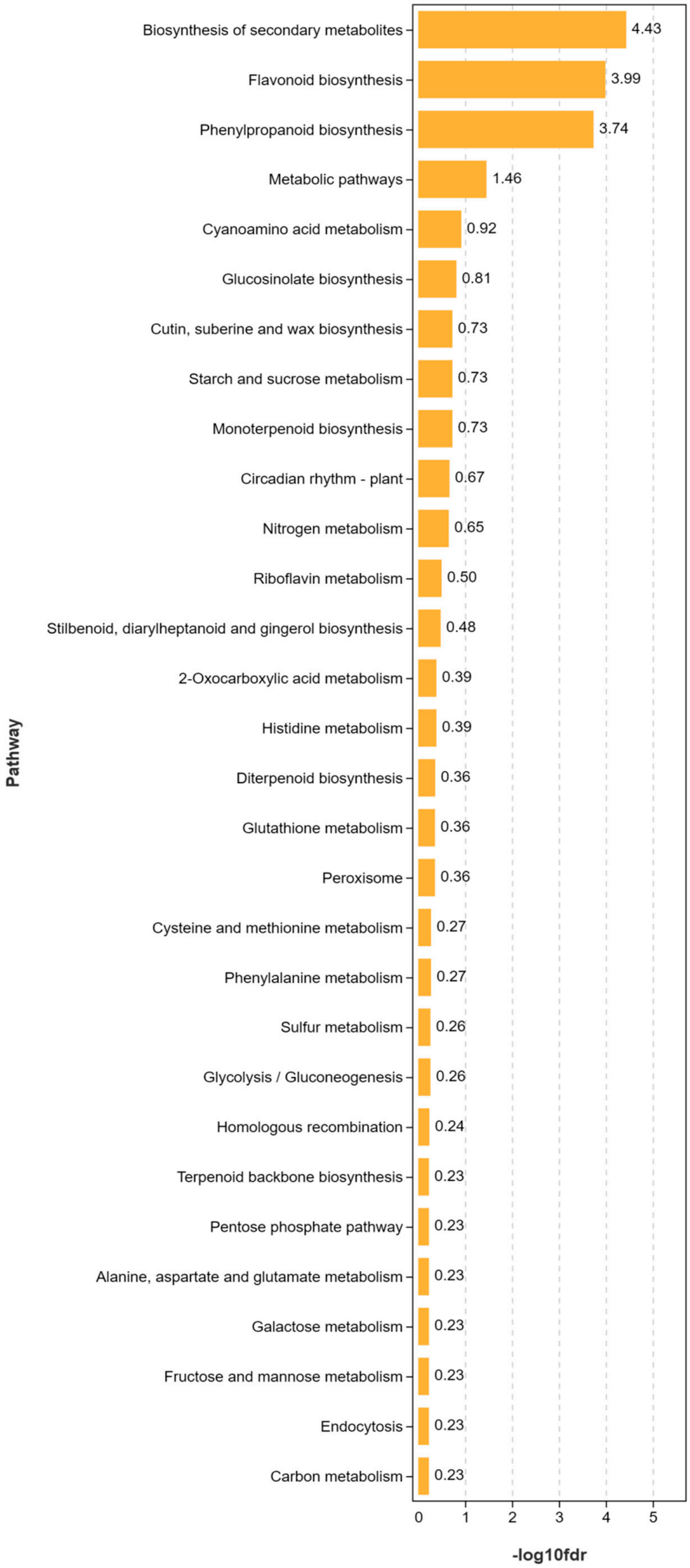

Biosynthesis of secondary metabolites
Flavonoid biosynthesis
Phenylpropanoid biosynthesis
Metabolic pathways
Cyanoamino acid metabolism
Glucosinolate biosynthesis
Cutin, suberine and wax biosynthesis
Starch and sucrose metabolism
Monoterpenoid biosynthesis
Circadian rhythm - plant
Nitrogen metabolism
Riboflavin metabolism
Stilbenoid, diarylheptanoid and gingerol biosynthesis
2-Oxocarboxylic acid metabolism
Histidine metabolism
Diterpenoid biosynthesis
Glutathione metabolism
Peroxisome
Cysteine and methionine metabolism
Phenylalanine metabolism
Sulfur metabolism
Glycolysis / Gluconeogenesis
Homologous recombination
Terpenoid backbone biosynthesis
Pentose phosphate pathway
Alanine, aspartate and glutamate metabolism
Galactose metabolism
Fructose and mannose metabolism
Endocytosis
Carbon metabolism
4.43
3.99
3.74
1.46
0.92
0.81
0.73
0.73
0.73
0.67
0.65
0.50
0.48
0.39
0.39
0.36
0.36
0.36
0.27
0.27
0.26
0.26
0.24
0.23
0.23
0.23
0.23
0.23
0.23
0.23
Pathway
0
1
2
3
4
5
-log10fdr

MM13.cyan

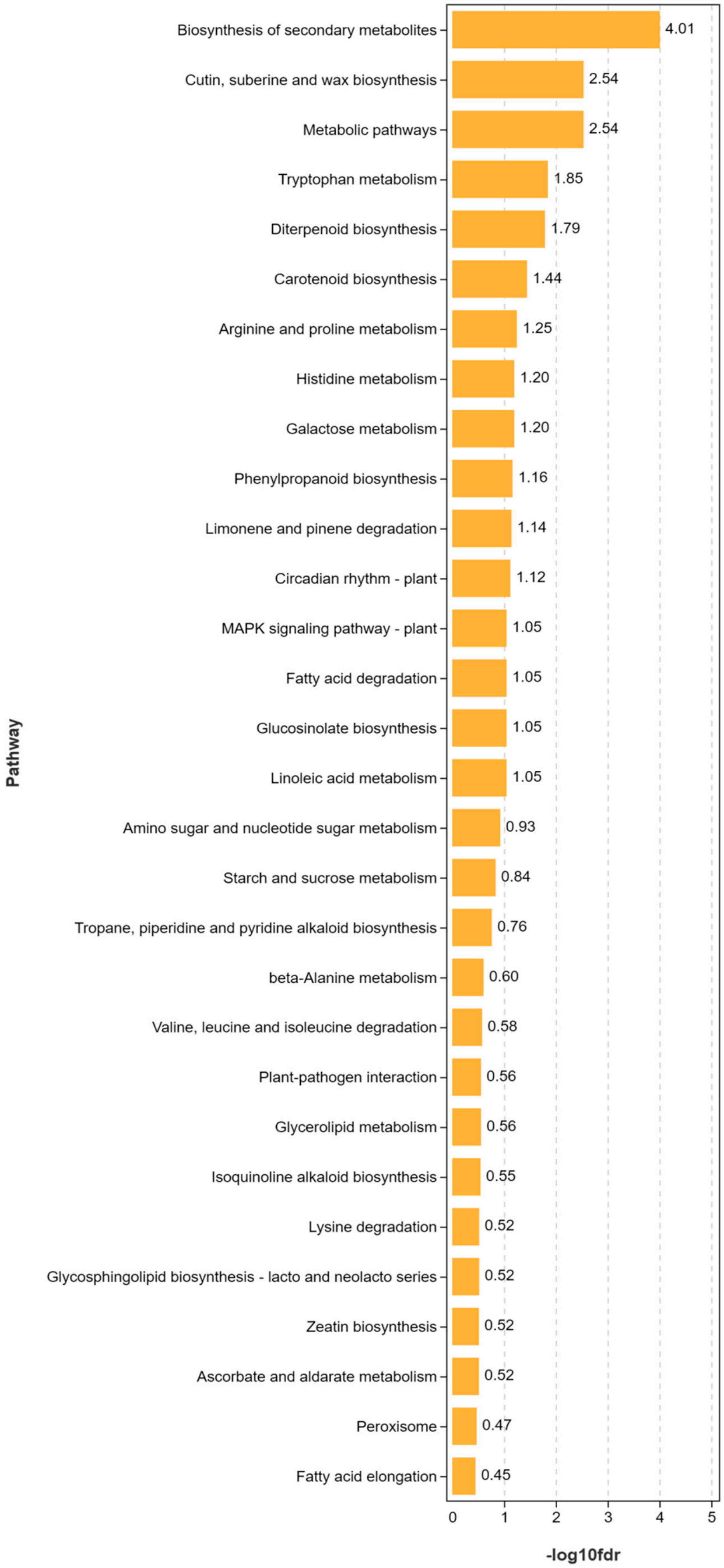

Pathway
-log10fdr
Biosynthesis of secondary metabolites 4.01
Cutin, suberine and wax biosynthesis 2.54
Metabolic pathways 2.54
Tryptophan metabolism 1.85
Diterpenoid biosynthesis 1.79
Carotenoid biosynthesis 1.44
Arginine and proline metabolism 1.25
Histidine metabolism 1.20
Galactose metabolism 1.20
Phenylpropanoid biosynthesis 1.16
Limonene and pinene degradation 1.14
Circadian rhythm - plant 1.12
MAPK signaling pathway - plant 1.05
Fatty acid degradation 1.05
Glucosinolate biosynthesis 1.05
Linoleic acid metabolism 1.05
Amino sugar and nucleotide sugar metabolism 0.93
Starch and sucrose metabolism 0.84
Tropane, piperidine and pyridine alkaloid biosynthesis 0.76
beta-Alanine metabolism 0.60
Valine, leucine and isoleucine degradation 0.58
Plant-pathogen interaction 0.56
Glycerolipid metabolism 0.56
Isoquinoline alkaloid biosynthesis 0.55
Lysine degradation 0.52
Glycosphingolipid biosynthesis - lacto and neolacto series 0.52
Zeatin biosynthesis 0.52
Ascorbate and aldarate metabolism 0.52
Peroxisome 0.47
Fatty acid elongation 0.45
0 1 2 3 4 5

MM14.tan

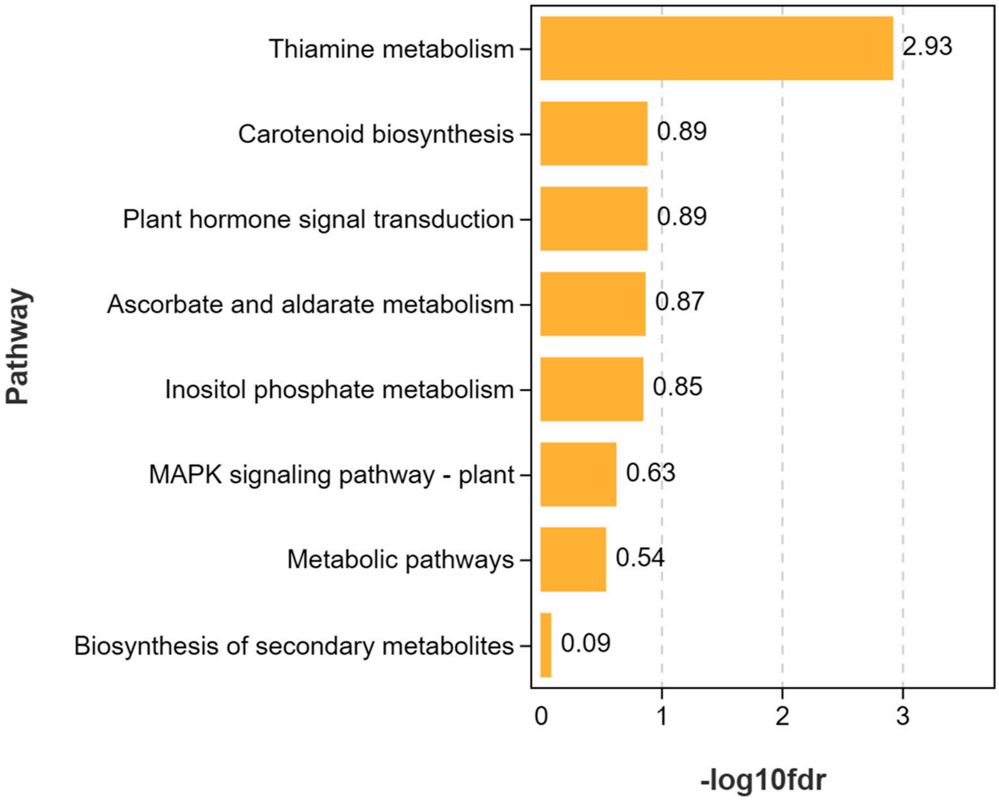

MM15.darkgrey

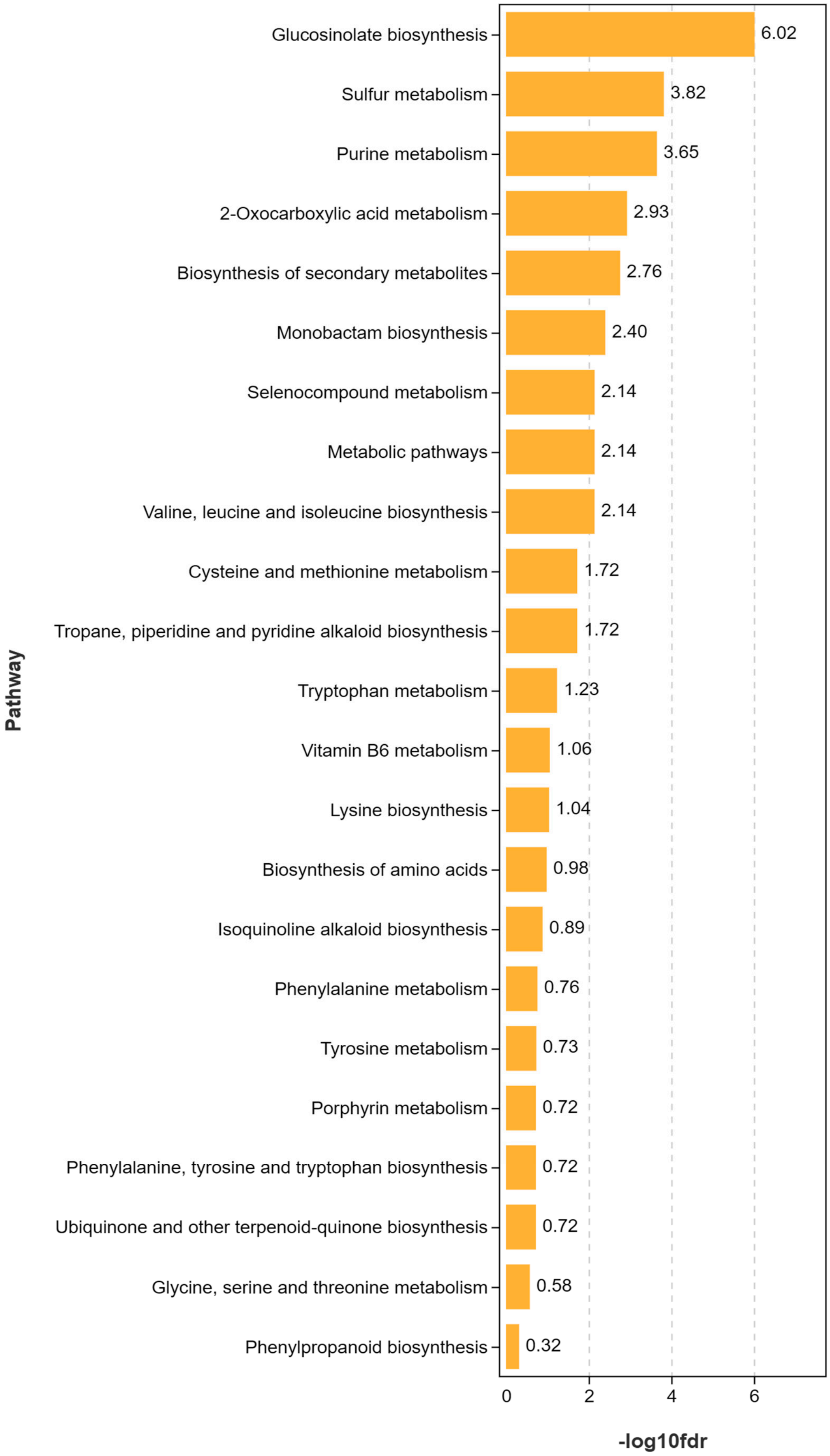

Glucosinolate biosynthesis
Sulfur metabolism
Purine metabolism
2-Oxocarboxylic acid metabolism
Biosynthesis of secondary metabolites
Monobactam biosynthesis
Selenocompound metabolism
Metabolic pathways
Valine, leucine and isoleucine biosynthesis
Cysteine and methionine metabolism
Tropane, piperidine and pyridine alkaloid biosynthesis
Tryptophan metabolism
Vitamin B6 metabolism
Lysine biosynthesis
Biosynthesis of amino acids
Isoquinoline alkaloid biosynthesis
Phenylalanine metabolism
Tyrosine metabolism
Porphyrin metabolism
Phenylalanine, tyrosine and tryptophan biosynthesis
Ubiquinone and other terpenoid-quinone biosynthesis
Glycine, serine and threonine metabolism
Phenylpropanoid biosynthesis
6.02
3.82
3.65
2.93
2.76
2.40
2.14
2.14
2.14
1.72
1.72
1.23
1.06
1.04
0.98
0.89
0.76
0.73
0.72
0.72
0.72
0.58
0.32
Pathway
0
2
4
6
-log10fdr

MM16.royalblue

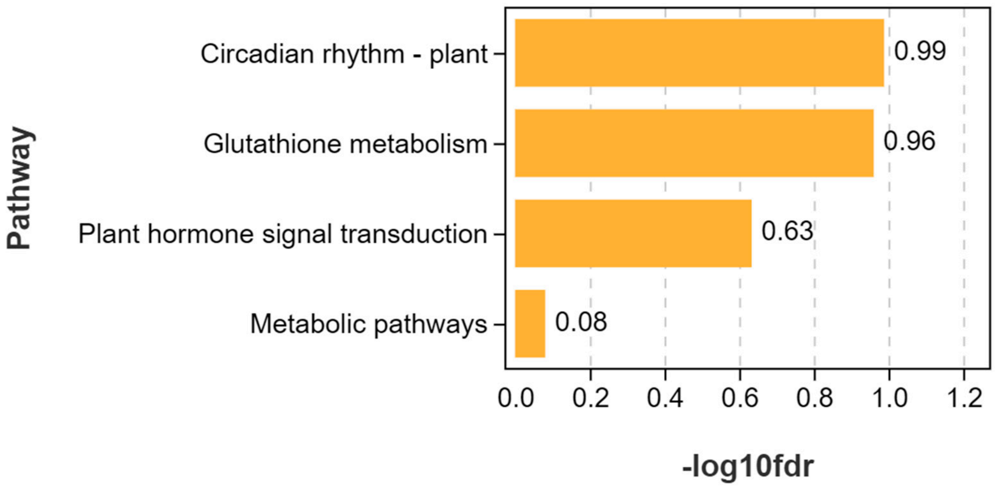

MM17.saddlebrown

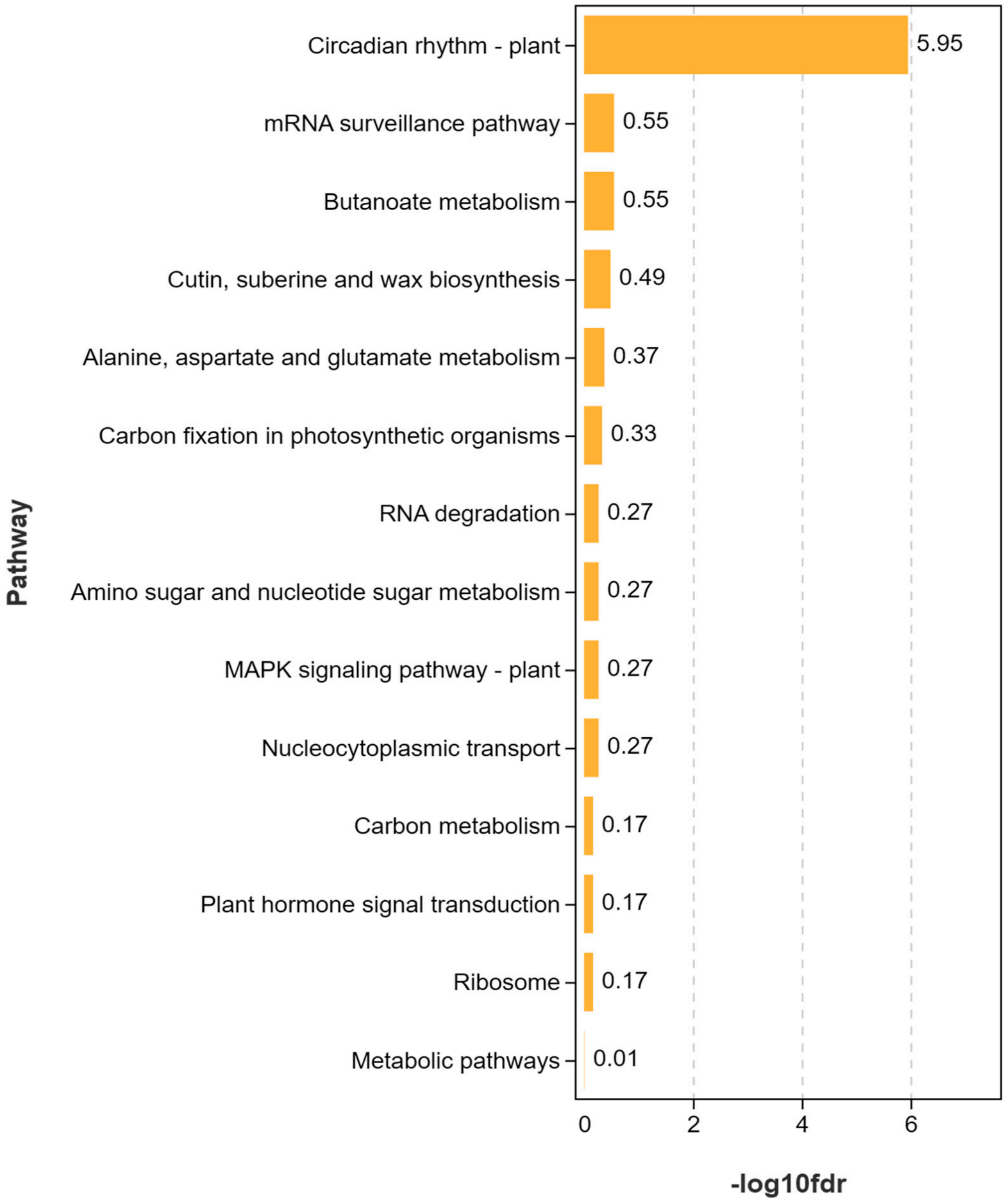
Circadian rhythm - plant
mRNA surveillance pathway
Butanoate metabolism
Cutin, suberine and wax biosynthesis
Alanine, aspartate and glutamate metabolism
Carbon fixation in photosynthetic organisms
RNA degradation
Amino sugar and nucleotide sugar metabolism
MAPK signaling pathway - plant
Nucleocytoplasmic transport
Carbon metabolism
Plant hormone signal transduction
Ribosome
Metabolic pathways
5.95
0.55
0.55
0.49
0.37
0.33
0.27
0.27
0.27
0.27
0.17
0.17
0.17
0.01
Pathway
0
2
4
6
-log10fdr

MM18.salmon
